# Human health risk assessment of selected trace elements in water–soil–vegetables system in Lephalale, Limpopo province, South Africa

**DOI:** 10.1016/j.toxrep.2026.102357

**Published:** 2026-10-15

**Authors:** Pertunia Ramolwetsi Dikotla, Abayneh Ataro Ambushe

**Affiliations:** Department of Chemical Sciences, Faculty of Science, University of Johannesburg, P.O. Box 524, Auckland Park, Johannesburg, 2006, South Africa

**Keywords:** Environmental contamination, Food safety, Human health risk, Mokolo River, Seasonal variation, Trace elements

## Abstract

The contamination of environmental systems by trace elements is an increasing concern in regions affected by intensive industrial and agricultural activities. These contaminants can infiltrate agricultural systems *via* irrigation with polluted water, resulting in food chain transfer and potential adverse effects on human health. In this study, water, sediment, soil, and vegetable samples were collected from the Mokolo River system in Lephalale, South Africa, and analysed for trace element concentrations. Samples were prepared, digested, and quantified using inductively coupled plasma-mass spectrometry (ICP-MS) and inductively coupled plasma-optical emission spectrometry (ICP-OES). To ensure accuracy of the analytical method, standard reference materials (SRMs), including trace elements in natural water standard reference material (SRM 1640a), tomato leaves standard reference material (SRM 1573a), trace elements in soil standard reference material (SRM 2587), and standard reference material of soil (AMIS 0373), were analysed, yielding percentage recoveries within acceptable ranges. Concentrations of trace elements ranged from 0.00120 to 13.5 µg/L in water, 0.0169–157 µg/g in sediment, 0.0890–94.1 µg/g in soil, and 0.00420–54.7 µg/g in vegetable samples. Elevated concentrations of nickel (Ni), lead (Pb), chromium (Cr), and cadmium (Cd) were observed, frequently exceeding established international guideline limits. Evaluation of human health risk assessment (HHRA) showed that Target Health Quotient (THQ) and Hazard Index (HI) values exceeded 1 for both adult and children, suggesting possible non-carcinogenic risks arising from dietary exposure. The carcinogenic risk assessment revealed elevated values of incremental lifetime cancer risk (ILCR), particularly for Cr and Pb, with consistently higher estimated risk in children than adults. These results underscore the risk of trace elements accumulation in the agricultural systems and its potential implications for human health. Continuous monitoring and appropriate management strategies are therefore recommended to mitigate contamination and reduce exposure risks.

## Highlights

• Contaminated irrigation water drives transfer of toxic metals into crops.
• Samples were prepared and mineralized using microwave-assisted acid digestion method.
• Eight toxic elements in water, soil and vegetables were quantified successfully.
• Validated methods were successfully applied to environmental samples from Lephalale.
• Human health risk assessment revealed non-carcinogenic health risks associated with dietary exposure.

## Graphical Abstract

**Figure:**
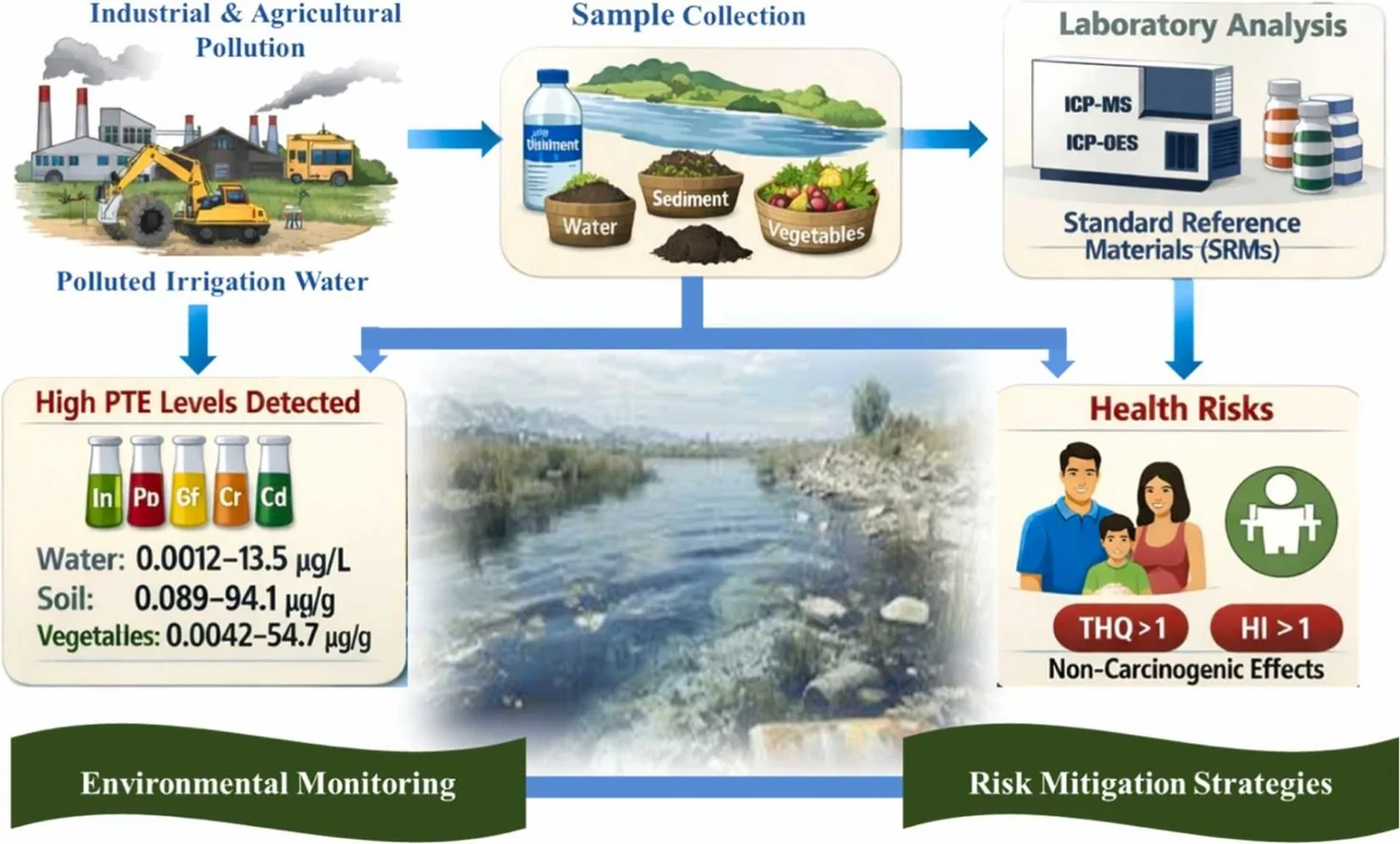

## 1 Introduction

Widespread contamination of environmental compartments; namely soil, water, and agricultural produce, by trace elements is a growing global concern [1]. These trace elements, which include arsenic (As), cadmium (Cd), chromium (Cr), copper (Cu), lead (Pb), mercury (Hg), nickel (Ni), and vanadium (V), are non-biodegradable and persist in ecosystems for extended periods [2], [3], [4]. Trace elements can originate from natural sources including the breakdown of mineral-rich rocks and volcanic activities. However, their elevated environmental concentrations are largely attributed to anthropogenic activities that include mining operations, industrial processes, fossil fuel combustion, and the intensive use of agrochemicals [5], [6], [7]. These activities are associated with the accumulation of trace elements in water and soil, leading to their transfer into the food chain and posing potential health risks through crop uptake and human consumption [8].

South Africa, like many developing nations, is experiencing increased industrialization, which contributes to elevated emission of trace elements into terrestrial and aquatic ecosystems [9]. Lephalale, located in the Limpopo province, is one such region affected by pollution due to its proximity to large-scale coal mining operations and the Matimba and Medupi coal-fired power stations. The Grootegeluk coal mine, in particular, is a major supplier of coal for the generation and production of electricity in the region [10], [11], [12]. The Mokolo River, which flows through this industrialized area, is a primary water source for both domestic consumption and agricultural irrigation [13]. Its location near coal-related activities increases its vulnerability to pollution through atmospheric deposition [14], surface runoff, and direct discharge of industrial effluents.

Previous studies have shown that irrigation with contaminated water can significantly increase the uptake of trace elements in vegetables, especially leafy crops, which tend to bioaccumulate trace elements in their tissues [15], [16], [17], [18]. Increased concentrations of Cr, Cd, As, and Pb have been reported in vegetables cultivated near industrial activities, with several studies indicating exceedances of safety limits established by the World Health Organization (WHO) and the Food and Agriculture Organization (FAO) [18], [19], [20]. Dietary consumption of trace element contaminated food represents an important pathway of human exposure. Prolonged exposure to As has been associated with neurological and cardiovascular effects and carcinogenicity, while Cd exposure can cause renal dysfunction and bone damage. Lead is of particular concern because of its adverse neurological and cognitive effects, particularly in children, whereas the toxicity of Cr depends on its chemical form, with Cr(VI) being more toxic and carcinogenic than Cr(III) [21], [22], [23]. Although Cu and Ni have biological functions at appropriate concentrations, excessive exposure may also results in adverse health effects [16], [24]. Consequently, chronic dietary exposure to trace elements through contaminated food may contribute to both non-carcinogenic and carcinogenic health risks.

Recent studies have demonstrated the importance of evaluating human health risks associated with dietary exposure to trace elements in plant derived food. Peirovi-Minaee et al. [25] assessed trace elements in processed grape products and reported age dependent differences in the estimated health risks, with children exhibiting greater vulnerability than adults. Similarly, Taghavi et al. [26] evaluated trace metals and metalloids in saffron and demonstrated the importance of considering both non-carcinogenic and carcinogenic risks associated with dietary exposure. More recently, Zarei et al. [27] investigated metal(oid)s in vegetables and reported potential health risks associated with vegetable consumption, further demonstrating the importance of vegetables as a dietary exposure pathway. These findings emphasise the need to evaluate trace element contamination in edible crops and the potential health implications for different population groups.

Although, studies in South Africa have investigated health risks linked with exposure to trace elements, pesticides and contaminated food, relatively few have applied the health risk assessment method to quantify potential risks from the consumption of the contaminated vegetables [16], [17], [20], [28]. A recent study by our group on As and Cr species in vegetables from the Lephalale area reported potential non-carcinogenic and carcinogenic risks associated with dietary exposure [10]. To date, the literature indicates that no integrated assessment has been conducted on trace elements across water, soil, sediment, and vegetable samples in this industrially impacted area.

The Lephalale region lacks a strong environmental monitoring framework for routine assessment of trace elements in vegetables and water, despite being highly susceptible to pollution. The continued expansion of mining and power generation activities, coupled with the reliance on river water for irrigation, necessitates a broader understanding of trace elements distribution across multiple environmental matrices. Monitoring the concentrations of trace elements in such regions is vital to safeguarding the environment and human health [14]. This study is among the first to comprehensively examine the seasonal variation and spatial distribution of eight (8) key trace elements across sediment, water, vegetables, and soil in the Mokolo River catchment, using validated analytical methods.

Recently, various analytical techniques have been applied for the quantification of trace elements in water, soil, and vegetable samples. Kapwata et al. [28] assessed trace elements contamination in household garden soils in rural Limpopo province, South Africa and quantified As, Pb, Hg, and Cd using X-ray fluorescence spectrometer (XRF). Similarly, Moyo et al. [17] assessed concentrations of Cd, Manganese (Mn), and Ni in fruits, vegetables and associated soil from Thohoyandou in Limpopo province, employing graphite furnace-atomic absorption spectrometry (GF-AAS). Although these previous methods have been successfully applied, the present study employed two advanced analytical techniques; namely, inductively coupled plasma-mass spectrometry (ICP-MS) and inductively coupled plasma-optical emission spectrometry (ICP-OES) for accurate determination of the target trace elements. These analytical techniques are commonly used due to their high sensitivity, allowing for the detection of trace elements across a variety of sample matrices [29]. An ICP-MS is highly sensitive and works well for detection of trace and ultra-trace levels of elements in water and vegetable samples, while ICP-OES is useful for soil and sediment because it handles more complex matrices [30]. These methods also allow testing of many trace elements at once and are reliable when validated with standard reference materials (SRMs).

Accordingly, this study aimed to assess concentrations of Cd, Cu, Hg, As, Pb, Cr, Ni, and V in environmental and agricultural samples collected from Lephalale. The specific objectives were to: (1) quantify concentrations of trace elements in water, sediment, soil, and vegetables using validated inductively coupled plasma-based techniques; (2) evaluate seasonal variation in trace elements distribution between low and high-flow seasons; and (3) evaluate non-carcinogenic and carcinogenic health risks associated with vegetable consumption using hazard index (HI), target health quotient (THQ), and incremental lifetime cancer risk (ILCR). It is hypothesized that the concentrations of trace elements in vegetables irrigated with Mokolo River water exceed international regulatory limits, thereby, posing potential health implications to consumers in the region. The outcomes of this study are intended to inform environmental policy, regulatory compliance, and agricultural safety in mining-impacted agroecosystems of South Africa.

## 2 Materials and methods

### 2.1 Reagents, standards, and certified reference materials

Analytical grade reagents were used throughout this study for the preparation of water, sediment, soil, and vegetable samples. The Suprapur 65% nitric acid (HN$O_{3}\;$) (Merck, Germany) was used for digestion of sediment, soil, and vegetable samples as well as for cleaning of ICP-MS cones. The 40% hydrofluoric acid (HF) (Merck, RSA), 65% HN$O_{3}$ and saturated boric acid $(H_{3}$B$O_{3}\;$) (Merck, Germany) were used for digestion of soil and sediment samples. Preparations of calibration standards were done daily, by diluting stock solutions of 1000 mg/L As (Inorganic ventures, USA), 1000 mg/L Cr(III) (PerkinElmer, USA), 1000 mg/L Cd(II) (Alfa Aesar A Johnson Matthey, USA), 1000 mg/L Cu(II) (Merck, RSA), 1000 mg/L of Pb(II) (Merck, RSA), 1000 mg/L Hg(II) (Merck, RSA), 1000 mg/L Ni(II) (Merck, RSA) and 1000 mg/L V(III) (Merck, RSA) appropriately. Internal standards were prepared using 1000 mg/L scandium (Sc(II)) (Merck, RSA), 1000 mg/L iridium (Ir(II)) (Merck, RSA) and 1000 mg/L rhodium (Rh(III)) (Merck, RSA) for ICP-MS analysis. Vegetable standard reference materials (SRMs), tomato leaves standard reference material (SRM-1573a) (National Institute of Standard Technology (NIST), USA), trace elements in soil standard reference material (SRM 2587) (NIST, USA), certified reference material of soil (AMIS 0373) (Transvaal Supergroup, RSA), and trace elements in natural water standard reference material (SRM 1640a) (NIST, USA) were used to optimize analytical procedures and verify the accuracy of methods for the analysis of vegetables, soil, sediment, and water samples, respectively.

### 2.2 Apparatus and instrumentation

Sample digestion was carried out using a One Touch MARS 6 microwave digestion system (CEM Corporation, USA). Samples were freeze-dried using a BK-FD10S freeze dryer (Biobase Bioindustry, China). The physio-chemical parameters of water samples were measured on-site using a HI 99100 multi-parameter meter. Quantification of analytes was done using a triple quadrupole NexION 300D ICP-MS (PerkinElmer, USA) for water and vegetable samples, while an ICPE-9800 ICP-OES (SHIMADZU, Japan) was employed for soil and sediment samples.

### 2.3 Sample collection, preservation, and storage

Sampling was carried out during two seasons to account for seasonal variation: August 2022 for the low-flow season and May 2023 for the high-flow season. Samples were collected from 15 sites during the low-flow season and 12 sites during the high-flow season, due to restricted access to some sites. These sites included points along the Mokolo River, Mokolo Dam, and three nearby farms. At each sampling site, approximately 1000 mL of water was collected into pre-cleaned, high-density polyethylene (HDPE) bottles, while sediment and soil samples were collected into 500 mL HDPE containers from a depth of 30 cm using an auger sampler. Immediately after sample collection, on-site measurements of pH were conducted using a portable multi-parameter meter. Water samples were subsequently kept in an insulated cooler with ice packs to maintain low temperatures during transport to the laboratory. Upon arrival at the laboratory, all water samples were filtered using 0.45 μm membrane filters (Merck, Darmstadt, Germany) to remove suspended solids. Filtered water samples were acidified with 1% Suprapur HNO₃ to prevent precipitation and microbial activity. All water samples were refrigerated at 4 °C prior analysis. Sediment and soil samples were air dried, before being crushed and homogenized. The material was then sieved using a 100 µm mesh to obtain a fine fraction suitable for digestion. Vegetables were washed with tap water to remove surface particulates and dust, followed by triple rinsing with de-ionized water. The samples were then cut into smaller pieces prior to freeze drying. After the freezing and drying process, the vegetable samples were crushed, homogenized, and passed through a 100 µm sieve to obtain a fine powdered sample prior to digestion.

### 2.4 Sample preparation

A portion of 200 mg of each vegetable sample, along with the tomato leaves SRM, was accurately weighed, and placed into Teflon digestion vessels. Approximately 6 mL of HNO₃ was added to each vessel, which was then left open for 5–10 min to allow the initial reaction to subside. Once the reaction settled, the vessels were properly sealed and loaded into the microwave digestion system (MARS 6, CEM Corporation). The digestion was carried out in a single stage as outlined: ramping to maximum power (1030–1800 W) over 20–25 min, followed by a 15-minute hold at a constant temperature of 200 °C, using 24 vessels. Soil and sediment samples, along with their respective SRMs, were prepared using a similar approach adopted from Mekonnen et al. [31] and Nsaka et al. [32]. A 100 mg portion of each sample was weighed into Teflon vessels. Digestion was carried out in two stages. In the first stage, 6 mL of HNO₃ and 0.5 mL of HF were added. The vessels were left open for 5–10 min before being sealed and subjected to microwave digestion following the same parameters used for vegetable samples. In the second stage, 1 mL of saturated H₃BO₃ was added to each vessel to neutralize excess fluoride, and digestion continued in the same microwave system. Saturated H₃BO₃ solution was freshly prepared by dissolving 7 g of H₃BO₃ in a 100 mL volumetric flask with deionized water. The solution was shaken, allowed to stand for complete dissolution, topped to the mark, and filtered through a 0.45 µm membrane filter [32].

Following digestion, all samples were allowed to cool to room temperature, quantitatively transferred into 50 mL volumetric flasks, diluted to the mark with deionized water, and filtered through 0.45 µm syringe filters before analysis. Ten reagent blanks for each matrix (vegetables, soil, and sediment) were prepared using the same procedures. All samples and blanks were prepared in triplicate to ensure analytical precision and reliability.

### 2.5 Sample analysis

The total concentrations of the selected trace elements in water and vegetable samples were determined using ICP-MS, while soil and sediment samples were analysed using ICP-OES. All analyses were performed under the optimized operational conditions outlined in Table 1 below.

**Table 1:** Instrumental operational conditions.

**ICP-MS**		**ICP-OES**	
**Component**	**Status**	**Component**	**Status**
Nebulizer gas flow	0.75 L/min	Argon Gas	491.92 P(kPa)
Auxiliary gas flow	1.4 L/min	Power	1.20 KW
Plasma gas flow	17 L/min	Plasma gas flow rate	14.00 L/min
ICP-RF power	1.5 KW	Auxiliary gas flow rate	1.20 L/min
Analogue stage voltage	-2818 V	Carrier gas flow rate	1.70 L/min
Pulse stage voltage	1550 V	Spectro Temperature	38.00 ℃
Torch box temperature	30.00 ℃	CCD Temperature	-14.00 ℃
Deflector voltage	-8.25 V	Vacuum level	51.5 Pa

### 2.6 Analytical figures of merit

To determine the limit of detection (LOD) for the analytical procedure, reagent blanks were prepared using the same procedure as the one applied for the quantification of trace elements total concentrations in the samples. The average of ten independently prepared reagent blank solutions was determined. The LOD for each trace element was calculated as three times the standard deviation (3σ) of the averaged reagent blank measurements. Similarly, the limit of quantification (LOQ) was calculated as ten times the standard deviation of the same averaged reagent blank measurements [12]. Different SRMs, including SRM 1573a, SRM 1640a, SRM 2587, and AMIS 0373, which contained varying concentrations of the selected trace elements were utilized to assess the preciseness of the analytical method. The accuracy of the procedure was assessed by comparing measured concentrations with certified values and calculating percentage recoveries. Method precision was determined by analysing all samples in triplicate and calculating the relative standard deviation (%RSD) of the corresponding triplicate readings.

### 2.7 Statistical analysis

Statistical analyses were performed using Microsoft Excel. Descriptive statistics, including means and standard deviations, were used to summarise the trace element concentration data. Seasonal differences between the low- and high-flow seasons were assessed using one-way analysis of variance (ANOVA), while one-sample *t*-tests were applied to compare mean concentrations of trace elements with the relevant guideline values. The normality of the datasets was assessed before interpretation of the parametric tests. Prior to interpretation of the one-sample *t*-test results, the normality of the concentration datasets was assessed using the Shapiro-Wilk test. Where significant departures from normality were identified, the corresponding parametric *t*-test results were interpreted cautiously and considered together with descriptive statistics and site-specific guideline exceedances. Statistical significance was evaluated at the 95% confidence level, with *p* < 0.05 considered statistically significant [11], [33], [34].

### 2.8 Human health risk assessment

Human health risk assessment (HHRA) was conducted to evaluate the potential non-carcinogenic and carcinogenic health risks associated with the consumption of the vegetable samples by adults and children.

#### 2.8.1 Non-carcinogenic risk

Non carcinogenic risks were assessed using the THQ and HI, calculated following (1), (2), (3). The HI was computed as the sum of the THQs of all analysed trace elements within each vegetable sample. The calculated THQ and HI values were interpreted using a threshold of 1, where values ≤ 1 indicate no significant non-carcinogenic health risk, whereas values > 1 indicate a potential non-carcinogenic health risk. The calculations were based on parameters shown in Table 2**,** adopted from Dintsi et al. [10] and Oladeji et al. [35].

(1) $$\mathrm{EDI}=\frac{\mathrm{Cmetal}\times \mathrm{IR}}{\mathrm{ABW}\times 1000}$$

(2) $$\mathrm{THQ}=\frac{\mathrm{EDI}}{\mathrm{RFD}}\;$$

(3) $$\mathrm{HI}=\sum_{i=1}^{n}\mathrm{TH}Q_{i}$$

**Table 2:** Parameters used for HHRA.

**Parameter**	**Symbol and units**	**Value**
Trace elements concentration	Cmetal (mg/kg)	-
Estimated daily intake	_EDI_ (year)	-
Ingestion rate	IR (g/day)	114.77 (Adult) 114.77 (children)
Oral reference dose	RfD (mg/kg/day)	0.003 (As), 0.0035 (Pb), 0.001 (Cd), 0.04 (Cu), 0.003 (Cr), 0.003 (Hg), 0.02 (Ni) and 0.007 (V)
Average body weight	ABW (kg)	68.1 (adult) 13.8 (children)
Oral slope factor	OSF	1.5 (As), 0.021 (Cr), 0.38 (Cd) and 0.0085 (Pb)

#### 2.8.2 Carcinogenic risk

The carcinogenic risk was determined following Eq. (4) and the Oral Slope Factor (OSF) are estimated for As, Cr, Cd, and Pb as shown in Table 2 [33]**.** The ILCR values exceeding ${1\;\times 10}^{-4}$are generally considered indicative of an elevated potential cancer risk, whereas values between ${1\;\times 10}^{-6}$ and ${1\;\times 10}^{-4}$ are typically regarded as falling within the acceptable or tolerable risk range for the exposed population.

(4) ILCR *=* EDI × OSF

## 3 Results and discussion

### 3.1 Analytical performance of the method

Linear calibrations curves were produced under optimum conditions for each analyte with coefficient of determination (R^2^) that ranged from 0.9996 to 1. The calibration curves showed excellent linearity and were considered acceptable [36]. SRMs appropriate for the different sample matrices were applied to assess the accuracy of the analytical methods used to quantify selected trace elements in water, sediment, soil, and vegetable samples using ICP-MS and ICP-OES. The measured and certified values of analytes in SRMs are presented in Table 3 below.

**Table 3:** Evaluation of the accuracy of methods.

Analyte	**SRM 1640a (µg/L)**		**SRM-1573a (ng/g**)		**AMIS-0373 & SRM-2587 (ng/g**)	
	Measured	Certified	Measured	Certified	Measured	Certified
As	9.30±0.10	8.075±0.070	0.10±0.005	0.113±0.002	78.7±1.53	83.6±14.6
Cd	3.80±0.17	3.992±0.074	1.46±0.073	1.52±0.027	1.51±0.01	1.80±0.4
Cr	41.2±0.26	40.54±0.30	1.79±0.055	1.99±0.034	88.7±3.08	109±31.1
Cu	88.6±0.63	85.75±0.51	4.75±0.730	4.70±0.14	16.1±0.327	20.0±8.3
Pb	11.8±0.23	12.101±0.050	1.36±0.026	1.78±0.24	17.5±1.35	20.9±4.0
Ni V	26.0±0.04 15.4±0.27	25.32±0.14 15.05±0.25	1.78±0.215 0.654±0.03	1.58±0.041 0.835±0.03	14.6±1.73 42.5±0.56	16.4±5.2 47.6±20.1

Overall, the percentage recoveries varied from 97.5% to 115% for trace elements in natural water (SRM 1640a), 76.4–113% in tomato leaves (SRM 1573a), 80.5–94.1% in SRM 2587 and AMIS 0373. The percentage recoveries of the specified analytes in water, sediment, soil and vegetable samples were within the acceptable range of 75–125% as suggested by US EPA [37]. Therefore, the obtained percentage recoveries validate the accuracy of the analytical methods used to quantify the selected trace elements in the specified sample matrices. The precision of the method was evaluated by determining the %RSDs from three consecutive runs of each sample. Lower %RSD that ranged from 0.0881% to 12.5% were obtained. The effectiveness of this method was proven by %RSD below 15%, falling within the acceptable range recommended by US EPA. This is consistent with the recommended %RSD values of less or equal for each analyte, thereby confirming the method’s reliability and reproducibility [38].

In this study, the LOD values obtained for the determination of selected trace elements in water, vegetable, sediment, and soil samples are summarized in Table 4. The LODs of Hg, V and Pb in water samples are comparable with those reported in the literature [32]. The determined Cr and Cu LOD’s were below 0.030 and 0.040 µg/L, respectively, as reported by the same authors. However, the LOD of Cd exceeded 0.0038 µg/L, as shown by the authors using ICP-MS. The LOD of As in this study is less than 0.22 µg/L as reported by Letsoalo et al. [12], with respect to ICP-MS employment. Alternatively, the LODs for vegetables, soil and sediment quantification methods, in this study, are lower than those presented in the literature [31] using ICP-MS, except for As, Cd, and V, which were slightly higher. Furthermore, the LOQs for water, soil, sediment and vegetables reported in this study are lower compared with those reported elsewhere [10]. The obtained high values of LOD and LOQ could be attributed to varied instrument conditions and reagents used. The LODs and LOQs observed in this study showed enhanced sensitivity of the ICP-MS and ICP-OES instruments used for analysis of samples.

**Table 4:** The LODs and LOQs for ICP-MS method (water and vegetables) and ICP-OES method (sediment and soil).

	**Water (µg/L)**		**Vegetable (ng/g)**		**Sediment & Soil (ng/g)**	
	LOD	LOQ	LOD	LOQ	LOD	LOQ
As	0.0198	0.0653	0.0334	0.110	0.267	0.881
Cd	0.0364	0.120	0.00951	0.0314	0.721	2.38
Cr	0.0114	0.0376	0.458	1.51	0.0351	0.116
Cu	0.0213	0.0703	0.226	0.746	0.0345	0.114
Pb	0.0477	0.157	0.0534	0.176	0.0752	0.248
Hg	0.00696	0.0230	0.0188	0.0620	0.0387	0.128
Ni V	0.00449 0.00720	0.0148 0.0218	0.175 0.0526	0.578 0.175	0.101 0.447	0.333 1.49

### 3.2 Quantification of total concentrations of trace elements in water

#### 3.2.1 The total concentrations of trace elements in water during low flow season

During low flow season, concentrations were 0.084 µg/L for V, 0.300 µg/L for Cr, 0.774 µg/L for Ni, 0.631 µg/L for Cu, 0.263 µg/L for As, 0.004 µg/L for Hg, and 0.004 µg/L for Pb (Fig. 1**)**. Overall, concentrations were relatively uniform across sampling locations, with no individual value exceeding the adopted guideline value.

**Fig. 1:**
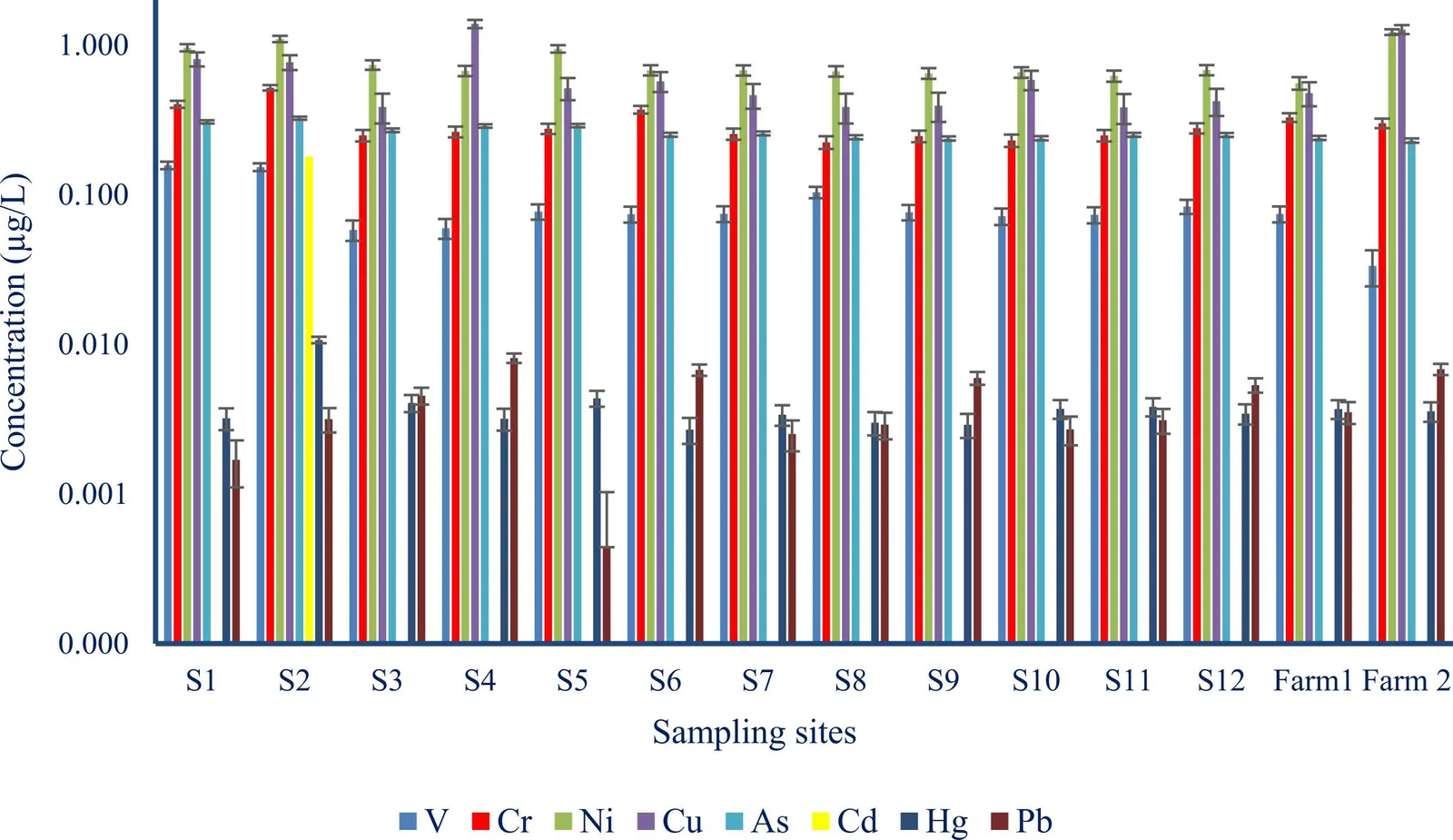
Total concentrations of trace elements in the water samples from Mokolo River during low flow season.

Cadmium was below the LOD of 0.0364 µg/L at most sampling sites and both farms, except for site 2, where a concentration of 0.180 µg/L was recorded. Agricultural activities in the vicinity of site 2 represent a potential local anthropogenic influence [39], [40]. However, the source of the detected Cd cannot be established from the present data. Comparable findings were reported by Nsaka et al. [32], who reported Cd concentrations below 0.0038 µg/L in most water samples analysed by ICP-MS, although a maximum concentration of 0.946 µg/L was recorded in the Wonderfonteinspruit Dam. The Cd concentration detected at site 2 in the present study was therefore lower than the maximum level reported by Nsaka et al. [32], while its occurrence at a single sampling site indicates spatial heterogeneity that warrants continued monitoring.

#### 3.2.2 The total concentrations of trace elements in water during high-flow season

Greater spatial variability was observed during high-flow season. Concentrations were 0.134 µg/L for V, 0.214 µg/L for Cr, 1.961 µg/L for Ni, 0.399 µg/L for Cu, 0.277 µg/L for As, 0.022 µg/L for Hg, and 4.341 µg/L for Pb (Fig. 2**)**. Lead concentrations reached 9.952 µg/L at site 1, 9.314 µg/L at site 5, and 8.900 µg/L at Farm 1, approaching the adopted drinking water guideline value of 10 µg/L. Nickel also showed a localised elevation of 13.508 µg/L at site 11.

**Fig. 2:**
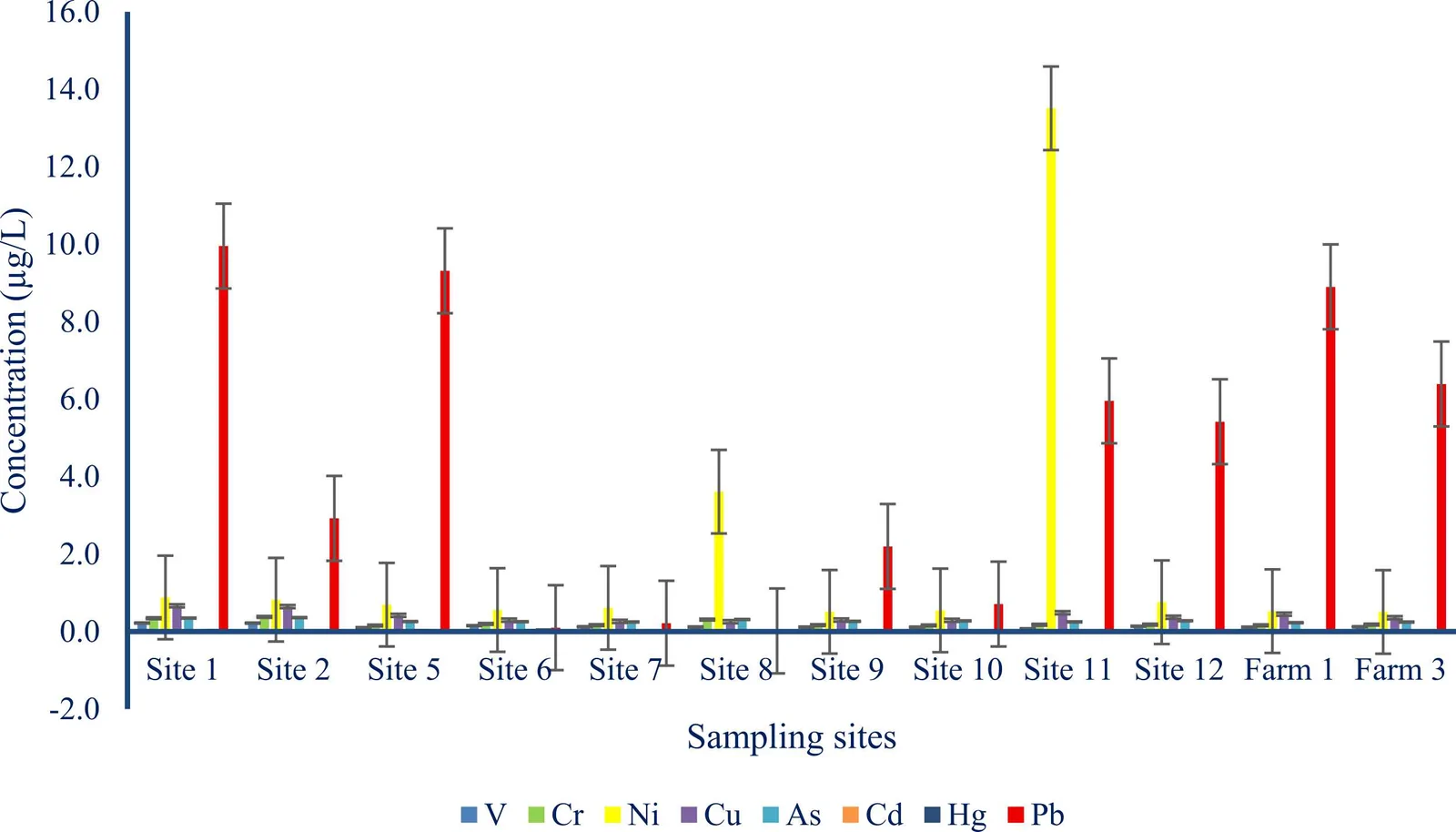
Total concentrations of trace elements in the water samples from Mokolo River during high flow season.

Nickel also showed spatial variability during high flow season, with the highest concentration of 13.5 µg/L recorded at site 11. Untreated industrial effluents and illegal dumping activities were observed in the vicinity of this sampling site, suggesting potential local anthropogenic influences. However, because source apportionment analysis was not conducted, these activities cannot be confirmed as the sources of the elevated Ni concentration. Similar spatial patterns have been reported by Shanbehzadeh et al. [41], who observed higher concentrations of trace elements downstream of sewage discharge points in the Tembi River, Iran. However, the Pb concentrations reported in their study reached 1950 µg/L downstream, substantially exceeding the maximum concentration of 9.95 µg/L recorded in the present study. This marked difference indicates a considerably lower magnitude of Pb contamination in the water samples from Mokolo River compared to the Tembi River study. However, direct comparison should be interpreted cautiously because of differences in pollution sources, catchment characteristics, hydrological conditions, sampling periods, and study design.

#### 3.2.3 Comparison of total concentrations of trace elements in water during low and high flow seasons

The total concentrations of all selected trace elements measured in water samples from the Mokolo River and Mokolo Dam during both low and high flow seasons were assessed against established guideline values for drinking and irrigation purposes. These guidelines serve to assess the water quality and ensure its viability for human intake and agricultural applications [42], [43], [44], [45]. Specifically, the maximum permissible levels (MPLs) outlined by the World Health Organization (WHO) [45], the South African National Standards (SANS) [44], and the Department of Water Affairs and Forestry (DWAF) [43] for drinking water were consulted. Additionally, the Target Water Quality Range (TWQR) established by DWAF [43] was applied to assess the adequacy of the water for assessment of agricultural irrigation. The applicable guideline values for drinking and irrigation water are presented in Table 5 and Table 6, respectively.

**Table 5:** The MPLs stipulated for drinking water.

Analyte	Guideline values			Maximum concentrations	
	WHO (µg/L)	DWAF (µg/L)	SANS (µg/L)	Low flow season	High flow season
As	10	10	10	0.325	0.362
Cd	3	5	3	0.180	0.0380
Cr	50	50	50	0.519	0.380
Cu	2000	1000	2000	1.39	0.665
Pb	10	10	10	0.00810	9.95
Hg	6	6	6	0.0107	0.0290
Ni	70	-	70	1.22	13.5
V	100	100	-	0.157	0.220

**Table 6:** The TWQR stipulated for irrigation water.

Analyte	Irrigation water guideline	Maximum concentrations	
	TWQR DWAF (µg/L)	Low flow season (µg/L)	High flow season (µg/L
As	100	0.325	0.326
Cd	10	0.180	0.0380
Cr	100	0.519	0.380
Cu	200	1.39	0.665
Pb	200	0.00810	9.95
Hg	-	-	-
Ni	200	1.22	13.5
V	100	0.157	0.220

One-sample *t*-tests confirmed that the mean concentrations of V, Cr, Ni, Cu, As, Hg, and Pb were significantly below their respective guideline values during both low and high flow season (*p* < 0.001). These findings provide statistical support for the concentration-based comparison and indicate that none of the investigated trace elements showed a group level exceedance of the adopted water quality guideline values set by DWAF, WHO, and SANS. However, the absence of a significant group level exceedance should not be interpreted as uniform compliance across all sampling locations, particularly where individual Pb concentrations approached the guideline value during high flow season.

A separate seasonal comparison revealed significant differences for Cd, Cr, Cu, Pb, Hg, and V (*p* < 0.05), whereas As and Ni did not differ significantly between sampling periods (*p* > 0.05). This demonstrates that seasonal behaviour was trace element-specific rather than characterised by a uniform increase or decrease across the river system.

The relatively low As concentrations measured in the present study are broadly consistent with previous findings from the Mokolo River. A recent study in Lephalale by Dintsi et al. [10] reported As concentrations of 0.168–0.274 µg/L during low flow season and 0.328–0.612 µg/L during high flow season. In comparison, As concentrations in the present study ranged from approximately 0.230–0.325 µg/L during low flow season and 0.229–0.362 µg/L during high flow season, indicating broadly comparable concentrations, although the previous study reported a higher high flow season maximum.

More recent study by Mamaregane et al. [46] reported substantially higher concentrations of several trace elements in the Mokolo River, with As, Cd, Cr, and Pb exceeding guideline values during the wet season. Their reported Pb concentrations of approximately 170–180 µg/L during the wet season and 270–800 µg/L during the dry season were markedly higher than the maximum Pb concentration of 9.952 µg/L observed in the present study. These differences highlight considerable spatial and temporal variability within the broader Mokolo River system. Direct comparison should nevertheless be made cautiously because the studies differed in sampling locations, sampling periods, environmental conditions, and analytical design.

Seasonal variation in river water chemistry may be influenced by changes in runoff, dilution, sediment-water interactions, and catchment inputs. Similarly, Mamaregane et al. [46] identified catchment geochemistry and surface runoff as possible contributors to higher wet season concentrations. However, because the present study did not perform source-apportionment analysis, mining, industrial activities, agricultural inputs, or wastewater discharge cannot be confirmed as the direct sources of the observed trace element patterns.

Therefore, the findings indicate that the investigated trace elements remained below the adopted water quality guideline values during both sampling periods, while localised and seasonal variability remained evident. From a practical perspective, monitoring should therefore be continued across different seasons and sampling locations, particularly at sites showing elevated Pb and Ni. The findings also demonstrate that compliance of river water with guideline values cannot be used alone to infer the absence of trace element contamination within the wider water-soil-vegetable system.

### 3.3 Quantification of total concentrations of trace elements in sediment

#### 3.3.1 The total concentrations of trace elements in sediment during low flow season

During low flow season, the greatest spatial variability was observed for Cd, Cr, and Cu. Chromium concentrations ranged from 7.67 to 69.33 µg/g and Cu from 2.27 to 47.08 µg/g, with the highest concentrations of both trace elements recorded at Site 9. Cadmium concentrations ranged from 0.64 to 6.45 µg/g and similarly reached their maximum at site 9 (Fig. 3**)**. The co-occurrence of elevated concentrations at this location suggests localised sediment enrichment. Site 9 is situated near a bridge or main road, where runoff, waste disposal, and sewage related activities were observed during sampling. These observations indicate potential local anthropogenic influences, although the contribution of individual sources cannot be established from the concentration data alone [47].

**Fig. 3:**
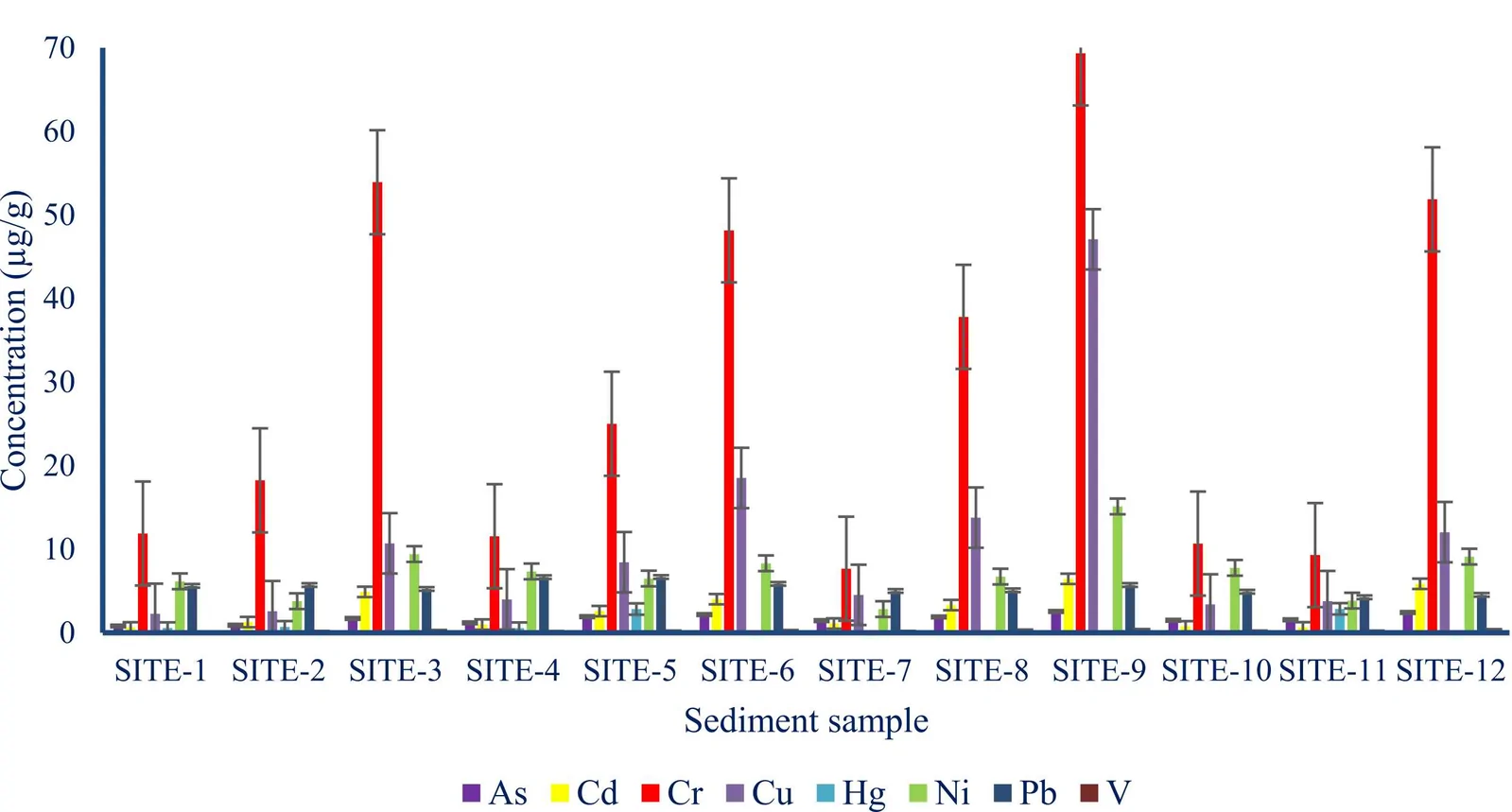
Total concentrations of analytes in sediment from Mokolo River and Dam during low flow season.

#### 3.3.2 The total concentrations of trace elements in sediment during high flow season

A distinctly different pattern emerged during high flow season, when Pb showed pronounced enrichment at sites 9 to site 12. Concentrations reached approximately 139, 157, 150, and 142 µg/g at Sites 9, 10, 11, and 12, respectively, compared with 5–7 µg/g at the other sampled sites (Fig. 4**).** The sharp spatial contrast indicates that Pb accumulation was localised rather than uniformly distributed throughout the river system. Such heterogeneity is consistent with the role of river sediments as sinks for particle associated trace elements, whose distribution can be influenced by local inputs, sediment characteristics, and depositional processes [48]. Furthermore, the sediment rich characteristics of the Mokolo River system suggest that both geogenic processes and anthropogenic inputs may contribute to the observed trace element concentrations [13], [17]. Addo-Bediako [48] similarly reported significant spatial differences in trace element concentrations among sediments from the Lower Olifants River Basin in Limpopo Province.

**Fig. 4:**
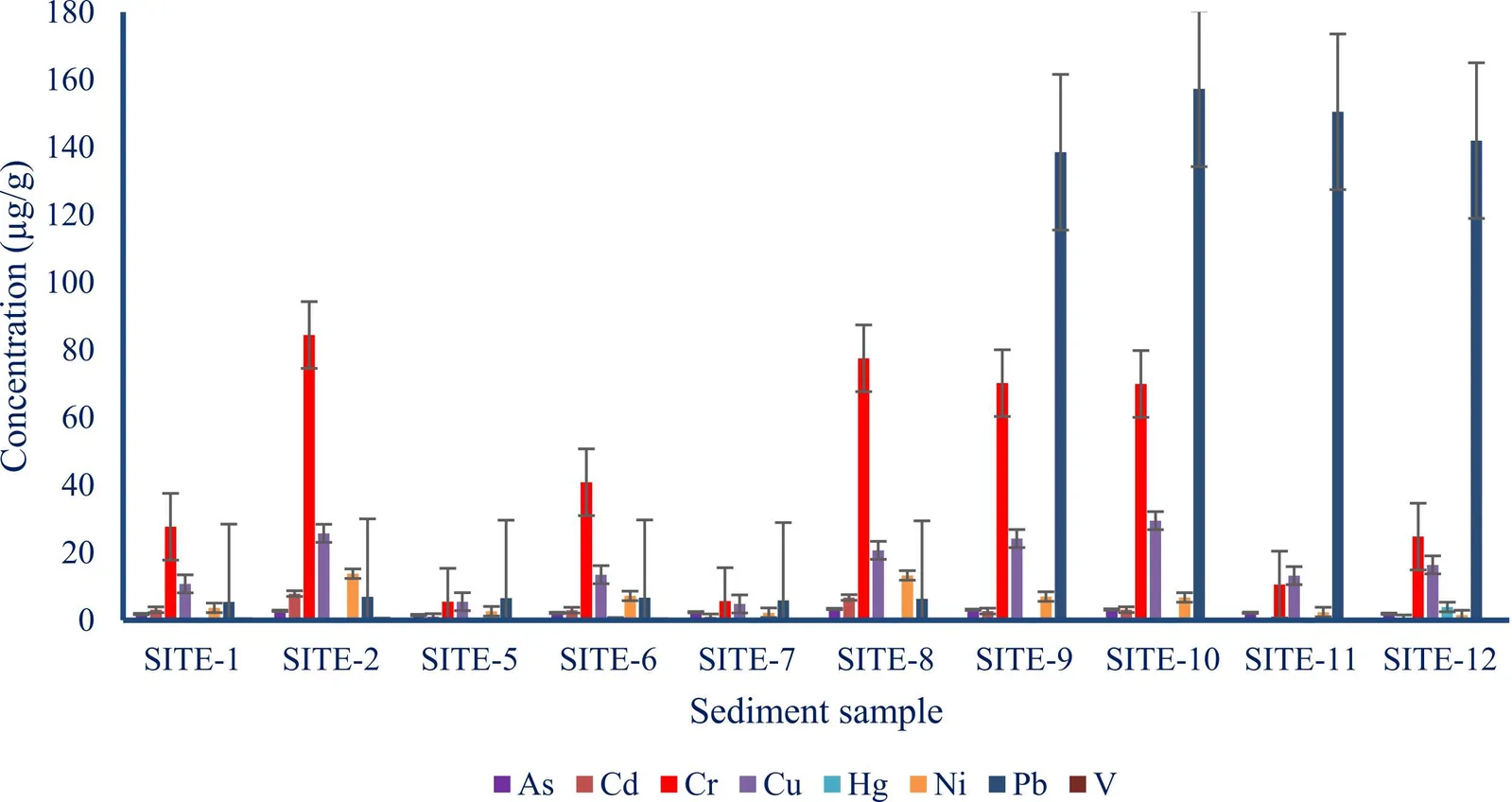
Total concentrations of trace elements in sediment from Mokolo River and Dam during high flow season.

#### 3.3.3 Comparison of trace elements total concentrations in sediment during low and high flow seasons

South Africa currently lacks established sediment quality benchmarks such as threshold effect concentration (TEC), probable effect levels (PEL), and interim sediment quality guidelines (ISQGs) for freshwater systems. In the absence of locally derived criteria, this study adopted the sediment quality guidelines developed by the Canadian Council of Ministers of the Environment (CCME) to assess potential ecological and human health risks associated with trace elements contamination in sediments. These guidelines are based on ecotoxicological, and ecological datasets compiled from multiple international studies and are therefore considered applicable in regions where national guidelines are not available. According to the CCME framework, sediment contaminant concentrations are interpreted in relative to three categories such as values below the ISQG, values between the ISQG and PEL, and values exceeding the PEL. Such variations may lead to different outcomes, including infrequent, occasional, or frequent adverse biological effects. The ISQG and PEL values for the selected trace elements examined in this study are presented in Table 7 [49], with the exception of Ni and V, as guidelines values for these trace elements are not available.

**Table 7:** The ISQGs and PELs for selected trace elements in sediment.

Analyte	Canadian Sediment Quality Guideline	
	ISQG (µg/g)	PEL (µg/g)
As	5.90	17.0
Cd	0.600	3.50
Cr	37.3	90.0
Cu	35.7	197
Pb	35.0	91.3
Hg	0.170	0.486
Ni	-	-
V	-	-

Statistical comparison with the CCME benchmarks further identified Cd as a consistent element of concern that exceeded sediment quality guideline. Concentrations of Cd during low flow season (2.714 µg/g) and high flow season (2.939 µg/g) were significantly greater than the ISQG of 0.600 µg/g (*p* = 0.006 and *p* = 0.018, respectively), although neither seasonal mean differed significantly from the PEL of 3.50 µg/g (*p* > 0.05). Importantly, individual Cd concentrations exceeded the PEL at sites 3, 6, 9, and 12 during this season and sites 2 and 8 during high flow season. This site specific pattern is ecologically important because concentrations exceeding the PEL are associated with an increased probability of adverse biological effects [49].

Chromium presented a less concerning pattern relative to the upper sediment quality benchmark. Mean concentrations of 29.61 µg/g during low flow season and 41.71 µg/g during high flow season did not differ significantly from the ISQG (*p* > 0.05) and remained significantly below the PEL of 90 µg/g (*p* < 0.001). Mean As and Cu concentrations were also below their respective benchmarks during both sampling periods, although Cu reached 47.08 µg/g at site 9 during this season, exceeding its ISQG of 35.7 µg/g. The Cr and Cu concentrations observed in the present study can be contextualised against other river sediments from Limpopo Province. Addo-Bediako [48] reported mean Cr concentrations of 44.6, 230.5, 40.3, and 863.9 mg/kg in the Blyde, Mohlapitsi, Ga-Selati, and Steelpoort Rivers, respectively, compared with seasonal means of 29.61 and 41.71 µg/g in the present study. Mean Cu concentrations in the Blyde, Mohlapitsi, and Steelpoort Rivers were 43.9, 14.6, and 15.1 mg/kg, respectively, compared with 10.93 and 16.43 µg/g during low and high flow season in the Mokolo River. The comparison demonstrates substantial variability among Limpopo River systems, particularly for Cr, which was markedly elevated in the Mohlapitsi and Steelpoort Rivers. Such differences may reflect variation in catchment geology, land use, sediment characteristics, and anthropogenic pressures among river systems rather than a uniform regional contamination pattern [48].

Lead warrants particular attention because its high-flow distribution differed substantially from the other investigated trace elements. Although the high flow season mean concentration (62.6 µg/g) did not differ significantly from either the ISQG or PEL (*p* > 0.05), individual concentrations at sites 9–12 exceeded the PEL of 91.3 µg/g. The lack of significant difference for the seasonal mean should therefore be interpreted cautiously because the Pb distribution was highly heterogeneous and departed from normality. The site specific Pb enrichment was also substantially greater than that reported by Addo-Bediako [48] for the Blyde River (4.9 mg/kg), Mohlapitsi River (6.1 mg/kg), and Steelpoort River (3.7 mg/kg). Thus, the concentrations of 139–157 µg/g recorded at sites 9–12 represent a notable localised feature of the Mokolo River sediments rather than a pattern generally observed across the Limpopo River systems considered here.

A direct comparison between seasons showed significant differences for As and Pb (*p* < 0.05), whereas Cd, Cr, Cu, Ni, and V did not differ significantly between low and high flow season (*p* > 0.05). The pronounced high-flow Pb enrichment may reflect changes in trace element mobilisation, particle transport, and sediment deposition associated with seasonal hydrological conditions. However, these processes were not measured directly and therefore remain plausible mechanisms rather than confirmed explanations for the observed seasonal pattern.

Collectively, the sediment results reveal contamination patterns that were less apparent from the water column, particularly the repeated Cd exceedances and pronounced high flow season Pb enrichment at sites 9–12. This supports the importance of sediment as an additional indicator of trace element contamination within the Mokolo River system.

The localised exceedances further identify specific areas requiring closer investigation, while future studies incorporating sediment physicochemical properties, trace element fractionation and source-apportionment approaches would help determine the mobility, bioavailability, and potential origins of the accumulated trace elements.

### 3.4 Quantification of trace elements total concentrations in vegetables

#### 3.4.1 The total concentrations of trace elements in vegetables during low flow season

During low-flow season, Cr concentrations ranged from 1.76 to 5.63 µg/g, with concentrations of several samples exceeding the MPL of 2.30 µg/g (Fig. 5). Vanadium concentrations ranged from 0.22 to 0.540 µg/g and exceeded the MPL of 0.030 µg/g in most samples. Selected vegetables also exceeded the respective MPLs for As, Cd, Hg, and Pb, whereas Ni and Cu remained below their respective MPLs of 10 µg/g in all low-flow samples. These results indicate that low-flow contamination was characterized primarily by widespread V and Cr exceedances, accompanied by localised exceedances of other trace elements.

**Fig. 5:**
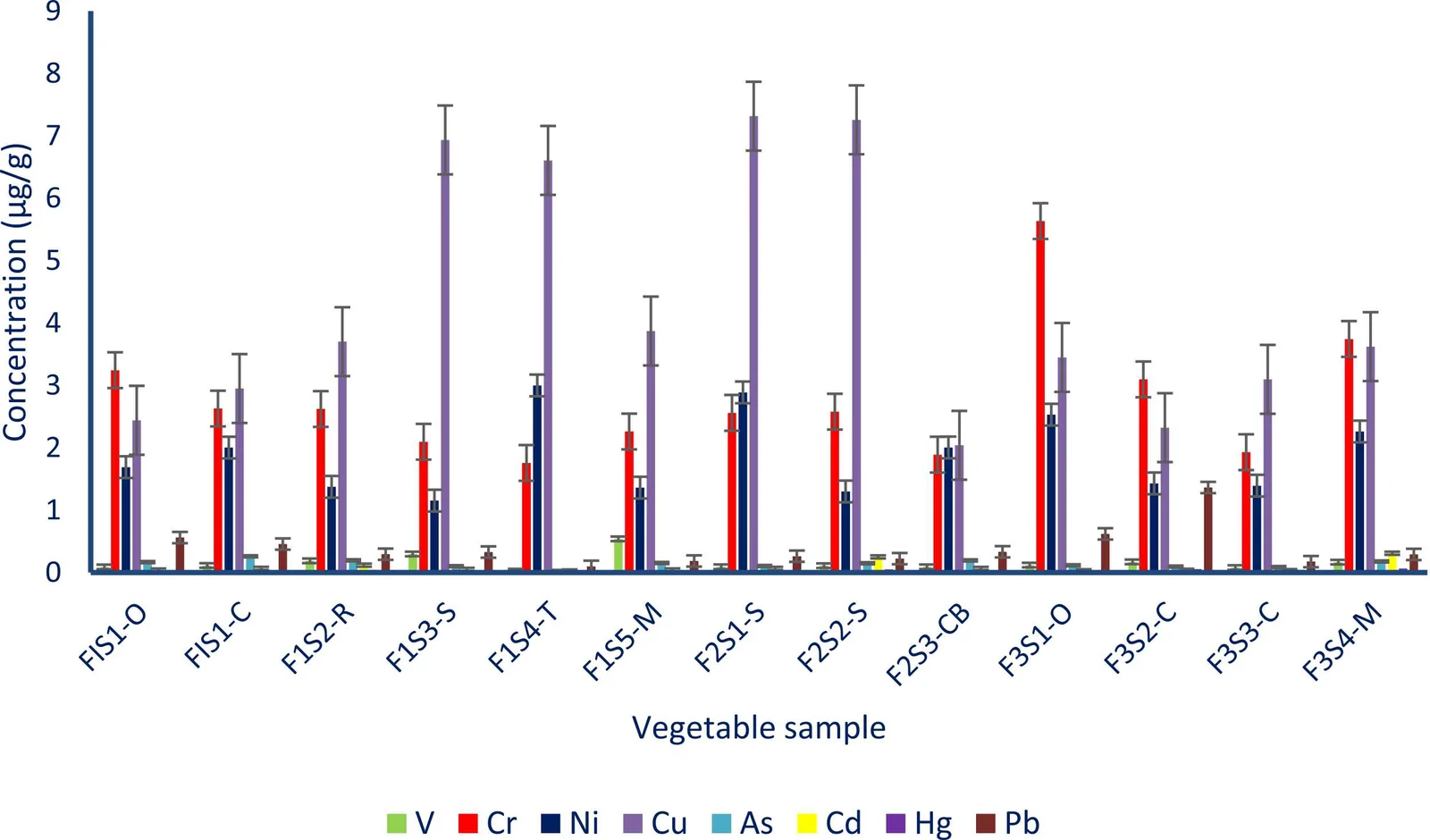
Total concentrations of trace elements in vegetables during low flow season.

#### 3.4.2 The total concentrations of trace elements in vegetables during high flow season

During the high flow season, seven vegetable samples were collected, and the total concentrations of all analysed trace elements in each sample are shown in Fig. 6.

**Fig. 6:**
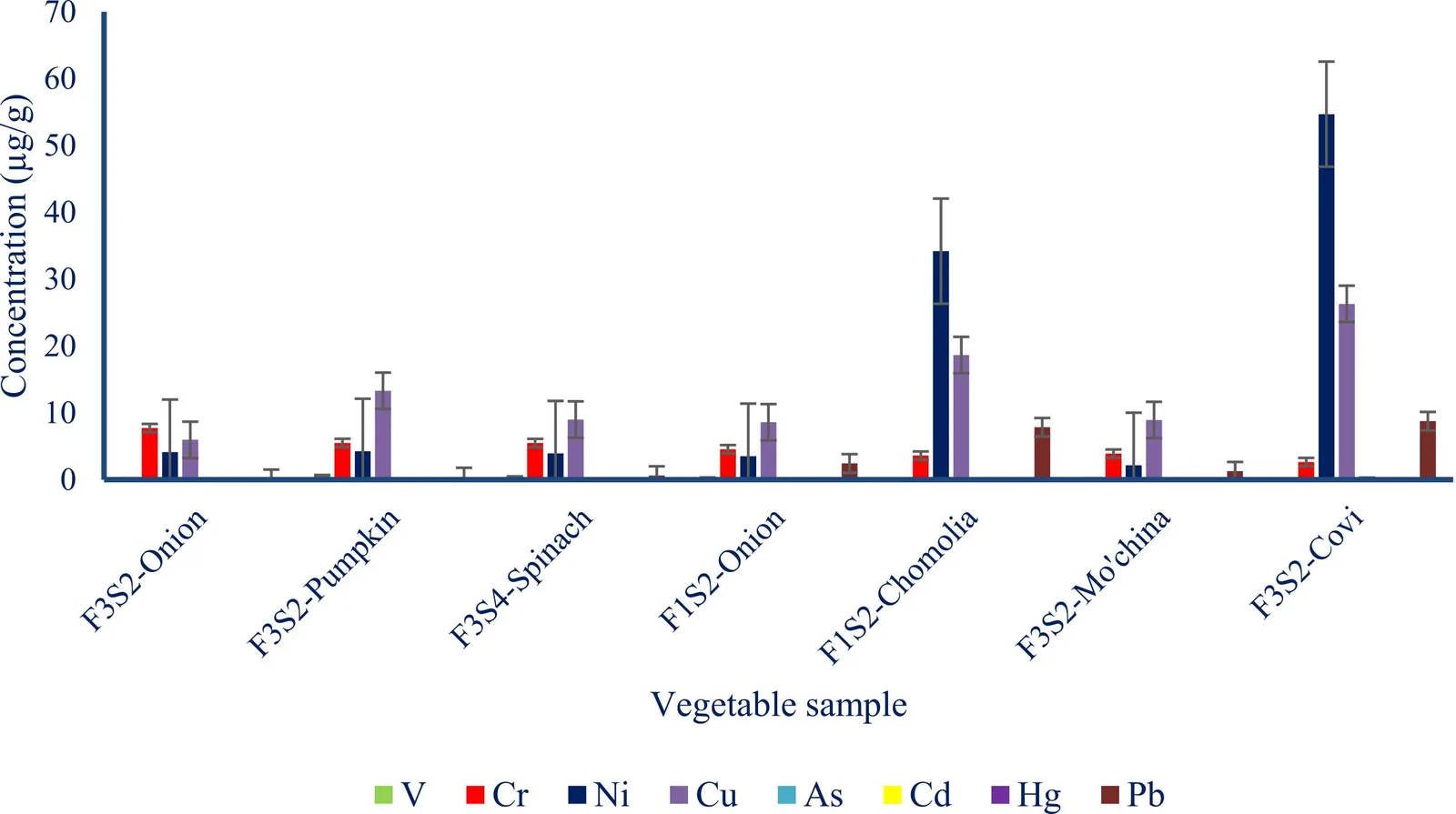
Total concentrations of trace elements in vegetables during high flow season.

A different concentration profile was observed during high flow season. Concentrations of Cr ranged from 2.63 to 7.72 µg/g and exceeded the MPL in all seven vegetable samples, while V similarly exceeded its MPL in all samples. Substantial sample specific elevations were also observed for Ni, Cu, and Pb. Nickel reached 34.18 µg/g in FIS2-Chomolia and 54.68 µg/g in F3S2-Covi, while Cu reached 18.64 µg/g and 26.30 µg/g in the same samples, respectively. Lead showed particularly elevated concentrations reaching 7.84 µg/g in FIS2-Chomolia and 8.75 µg/g in F3S2-Covi, compared with an MPL of 0.30 µg/g. In contrast, Cd and Hg remained below their respective MPLs in all high-flow samples. Therefore, these results indicate that high-flow contamination was characterised by widespread V and Cr exceedances together with substantial enrichment of Ni, Cu, and Pb in particular vegetables.

The occurrence of elevated concentrations in edible vegetables is consistent with findings reported in other studies. Gupta et al. [50] identified vegetables as important accumulators of trace elements and highlighted that accumulation and translocation are influenced by both plant related and environmental compartments. Similarly, Alsafran et al. [51] reported elevated concentrations of V, Cr, As, Cd, and Pb in leafy vegetables, with several concentrations exceeding the recommended MPL. A study by Basha et al. [52] also reported a Cr concentration of 1.0–9.7 mg/kg in vegetables and fruits cultivated around a uranium mining area, demonstrating substantial variability in Cr accumulation among edible crops. These comparisons indicate that elevated trace element concentrations in vegetables are not unique in the present study. However, direct qualitative comparisons should be made cautiously because of differences in crop species, environmental conditions, analytical approaches and local contamination profiles.

Overall, the vegetable samples collected from farms surrounding the Mokolo River during low and high flow seasons exhibited a range of trace element concentrations. These results were compared with the MPLs established by the WHO/FAO, as well as with findings from related studies (Table 8) [31], [53], [54], [55], [56].

**Table 8:** The MPLs stipulated by WHO/FAO in vegetables.

Analyte	Guideline	Maximum Concentrations	
	WHO & FAO (µg/g)	Low flow season (µg/g)	High flow season (µg/g)
As	0.1 [53], [54], [55]	0.260	0.273
Cd	0.2 [53], [55], [56]	0.309	0.0825
Cr	2.3 [53], [57]	5.63	7.72
Cu	10 [53], [54], [56]	7.31	26.3
Pb	0.3 [53], [55], [56]	1.36	8.75
Hg	0.03 [53], [58]	0.0637	0.0255
Ni	10 [53], [56]	3.00	54.7
V	0.03	0.540	0.630

To complement the sample specific comparison with MPLs, one sample *t*-test were used to determine whether the mean concentration of each trace element differed significantly from its corresponding recommended MPLs. During low flow season, the mean concentrations of V (0.160 µg/g) and As (0.140 µg/g) were significantly greater than their respective MPLs of 0.030 and 0.100 µg/g, with *t*(12) = 3.55, *p* = 0.004 and *t*(12) = 2.40, *p* = 0.034, respectively. Although the mean concentration of Cr (2.772 ± µg/g), Hg (0.036 µg/g), and Pb (0.399 µg/g) were numerically above their respective MPLs. These differences were not statistically significant (*p* > 0.05). Conversely, the mean concentration of Ni, Cu, and Cd were significantly below their corresponding MPLs (*p* < 0.001).

During high flow season, V remained significantly above its MPL, with a mean concentration of 0.263 µg/g, *t*(6) = 3.12, *p* = 0.021. Chromium showed a stronger group level exceedance during this sampling period as its mean concentration increased to 4.76 µg/g and was significantly greater than the MPL, *t*(6) = 3.93, *p* = 0.008. Although the mean concentration of Ni (15.3 µg/g), Cu (13.0 ± µg/g), and Pb (3.06 µg/g) were numerically greater than their corresponding MPLs, the differences were not statistically significant (*p* > 0.05). In contrast, mean Cd and Hg concentrations were significantly below their respective MPLs (*p* < 0.001), while As did not differ significantly from its MPL (*p* = 0.504).

The statistical analysis therefore revealed distinct element specific patterns between the two sampling periods. Vanadium was the only trace element for which the mean seasonal concentration significantly exceeded the MPL (Table 8) during both low and high flow seasons, indicating a persistent group level exceedance. Chromium showed a different pattern, although its high-flow mean concentration was numerically above the MPL, the difference was not statistically significant, whereas its high flow season concentration significantly exceeded the MPL. Conversely, As showed a significant group level exceedance during low flow season, but not during high flow season. However, these findings should not be interpreted as demonstrating statistically significant difference between two seasons, because the one-sample *t*-test compared each seasonal mean with MPLs established by WHO/FAO rather than directly comparing low and high flow season concentrations. A separate statistical comparison between the two sampling periods showed significant seasonal differences in the concentrations of Cr, Cu, Pb, Hg, and Ni (*p* < 0.05), whereas As, Cd, and V did not differ significantly between the low- and high-flow periods (*p* > 0.05) [10].

The elevated Cr concentrations observed in the present study are comparable with previous findings from vegetables cultivated in Lephalale. Dintsi et al. [10] reported total Cr concentrations ranging from approximately 1.02–4.15 µg/g during low flow season and 2.58–18.4 µg/g during high flow season in vegetables collected within the same broader study area. In comparison, Cr concentrations in the present study ranged from 1.76 to 5.63 µg/g during low flow season and 2.63–7.72 µg/g during high flow season. Thus, the concentration ranges were broadly comparable, although Dintsi et al. [10] reported a considerably higher maximum concentration during high flow season. The occurrence of higher Cr concentrations during high flow season in both studies provides supporting evidence of a similar seasonal pattern within the Lephalale area. However, additional multi season investigations would be required to determine whether this pattern is consistent over time.

The variability observed among individual vegetables is consistent with previous evidence that trace element accumulation in edible crops is influenced by plant species and environmental conditions. Gupta et al. [50] reported that bioavailability and accumulation at the soil-vegetable interface are influenced by soil and plant related factors and emphasised that leafy vegetables may accumulate comparatively high concentrations of trace elements. Alsafran et al. [51], similarly observed differences in V, Cr, Ni, Cu, As, Cd, and Pb concentrations among leafy vegetables species, demonstrating that accumulation is not uniform among the crops. More recently, Zarei et al. [27] reported considerable variations in 11 vegetable species and found that As, Cd, Ni and Pb exceeded WHO/FAO MPLs. These findings support the crop specific variability observed in the present study. However, because bioavailable soil fractions, crop specific uptake mechanisms and transfer factors were not determined in the present study, the observed differences among vegetables cannot be attributed to a particular mechanism.

The Cr findings warrant particular attention. During high flow season, all seven vegetable samples exceeded the Cr MPL, and concentration was significantly greater than the MPL. Comparable evidence of Cr accumulation in edible crops has been reported elsewhere [52]. Trace element concentrations in vegetables and fruits cultivated around the Tummalapalle uranium mining area ranged up to 9.7 mg/kg for Cr. Similarly, a South African study of plant leaves from a mining smelting region by Adhikari et al. [59] reported total Cr concentrations exceeding MPL and identified Cr as an important contributor to estimated health risks. These findings provide broader evidence that elevated Cr can occur in edible or useful plant tissues in mining influenced environments. However, the present findings should not be interpreted as demonstrating a mining source for Cr because source apportionment was not undertaken. Furthermore, the present study quantified total Cr rather than individual Cr species. Therefore, the measured concentrations cannot be attributed specifically to Cr(VI) or Cr(III) without speciation analysis.

The Pb results further demonstrate why statistical comparisons of seasonal means should be interpreted alongside individual sample concentrations. During high flow season, the mean Pb concentration (3.06 µg/g) was more than ten times the MPL of 0.300 µg/g, but the difference was not statistically significant (*p* = 0.093). This result occurred in the presence of substantial variability, with Pb concentrations ranging from 0.122 µg/g in F3S2-Onion to 8.75 µg/g in F3S2-Covi. During low flow season, the mean Pb concentration was also numerically above the MPL but did not differ significantly from it (*p* = 0.295). Therefore, the absence of statistical significance should not be interpreted as evidence that all vegetables complied with the MPL value. Zarei et al. [27] similarly reported vegetable dependent Pb concentrations ranging from 0.18 to 2.47 mg/kg, illustrating considerable variability in Pb accumulation among vegetable species. The pronounced concentrations observed in F1S2-Chomolia and F3S2-Covi are particularly relevant because these samples subsequently produced some of the highest Pb related ILCR estimates in the health risk assessment.

The elevated Ni and Cu concentrations in selected high flow season vegetables showed a similarly heterogeneous pattern. Although their high flow season concentrations were above the corresponding MPLs, neither difference was statistically significant, largely reflecting the substantial variation among individual samples. Crop specific variation in Ni and Cu has also been observed elsewhere. For example, Zarei et al. [27] reported Ni concentrations ranging from 0.19 to 4.51 mg/kg and Cu from 5.25 to 13.58 mg/kg among different vegetable species. The Cu concentrations observed in this study, which ranged from 2.04 to 7.31 µg/g, fall within the same range. The considerably higher Ni concentrations recorded in F1S2-Chomolia and F3S2-Covi in the present study therefore identify these samples as noteworthy observations requiring consideration independently of the non-significant group-level comparison.

Considerable variation in trace element concentrations among vegetables has similarly been documented in South Africa. A Johannesburg study of leafy vegetables by Adhikari and Struwig [60] reported concentrations across vegetable groups ranging from approximately 7.79–74.3 mg/kg for Cr, 8.00–199 mg/kg for Ni, 34.3–46.5 mg/kg for Cu, 0.360–2.56 mg/kg for As, and 0.120–2.45 mg/kg for Hg on a dry weight basis. Although concentrations in the present study were generally lower for several of these trace elements, substantial Ni concentrations were observed during high flow season, particularly in F1S2-Chomolia (34.2 µg/g) and F3S2-Covi (54.7 µg/g). The considerable variability reported in both studies supports the importance of evaluating trace element accumulation at the level of individual vegetable types rather than relying exclusively on aggregated concentrations. Bvenura and Afolayan [61] reported the highest Cu concentrations in onion, followed by tomato and spinach, with the lowest concentrations observed in cabbage. In contrast, the present study showed a different distribution, with spinach exhibiting the highest Cu concentrations, followed by tomato, while cabbage contained the lowest concentrations. Such differences demonstrate that Cu accumulation may vary considerably among vegetable types and across study areas. Previous studies have suggested that leafy vegetables may accumulate relatively high concentrations of some trace elements compared with other vegetable groups [31], although accumulation is dependent on the trace element, vegetable species, and prevailing environmental conditions. Similar variability in trace element concentrations among vegetable species has been reported by Moyo et al. [17] and Kapwata et al. [28] in vegetable samples from Limpopo Province.

The combined results also demonstrate that compliance of irrigation water with guideline values does not necessarily establish the safety of vegetables cultivated within the same agricultural system. Accumulation of trace elements in vegetables can be influenced by multiple environmental and plant related factors, and Gupta et al. [50] consequently emphasised the importance of considering trace element concentrations in irrigation water and soil when seeking to minimise contamination of vegetables. Similarly, studies evaluating contaminated leafy vegetables have recommended monitoring trace elements across relevant environmental and food chain compartments rather than relying solely on measurements at the point of consumption [16], [51]. Nevertheless, the present study did not establish direct transfer relationships among river water, agricultural soils, and vegetables. Consequently, the concentrations detected in vegetables cannot be attributed exclusively to either irrigation water or soil contamination.

From a practical perspective, the findings support an integrated approach to environmental and food safety monitoring within the Mokolo River agricultural system. Attention is warranted for V and Cr because of their widespread exceedances, while the pronounced sample-specific concentrations of Pb, Ni, and Cu demonstrate that seasonal concentrations alone may obscure contamination affecting particular crops or sampling locations. Recent research has similarly recommended routine monitoring of trace elements in vegetables where concentration exceedances and associated dietary health risks occur [27]. Monitoring edible vegetables alongside agricultural soils and irrigation water would therefore provide a more comprehensive basis for identifying potential exposure concerns, prioritising affected locations and crops, and informing subsequent investigations of trace element bioavailability and soil-to-plant transfer.

### 3.5 Quantification of trace elements total concentrations in soil

#### 3.5.1 The total concentrations of trace elements in soil during low flow season

During the low flow season, Cd concentrations ranged from 2.92 to 8.15 µg/g, while Cr concentrations ranged from 34.8 to 94.1 µg/g. The highest Cr concentrations were recorded at F2S1-S (92.9 µg/g) and F2S2-S (94.1 µg/g). Copper showed considerable spatial variation, ranging from 11.7 to 65.6 µg/g, with the highest concentration recorded at FIS2-S (Fig. 7**)**. In comparison, As, Ni and Pb revealed relatively narrower concentration ranges across the sampled agricultural soils. These results demonstrate that the distribution of trace elements during low flow season was element and site specific, with Cd occurring consistently across the sampled soils and localised elevations evident for Cr and Cu. Similar variability in the trace element occurrence within South African soil-crop systems have been reported by Mngadi et al. [62], who investigated soils and *Brassica oleracea* near an abandoned gold-mine dump in Johannesburg, demonstrated the occurrence of both toxic and essential trace elements across the soil-vegetable system and highlighted the importance of considering agricultural soil together with edible crops when evaluating potential exposure.

**Fig. 7:**
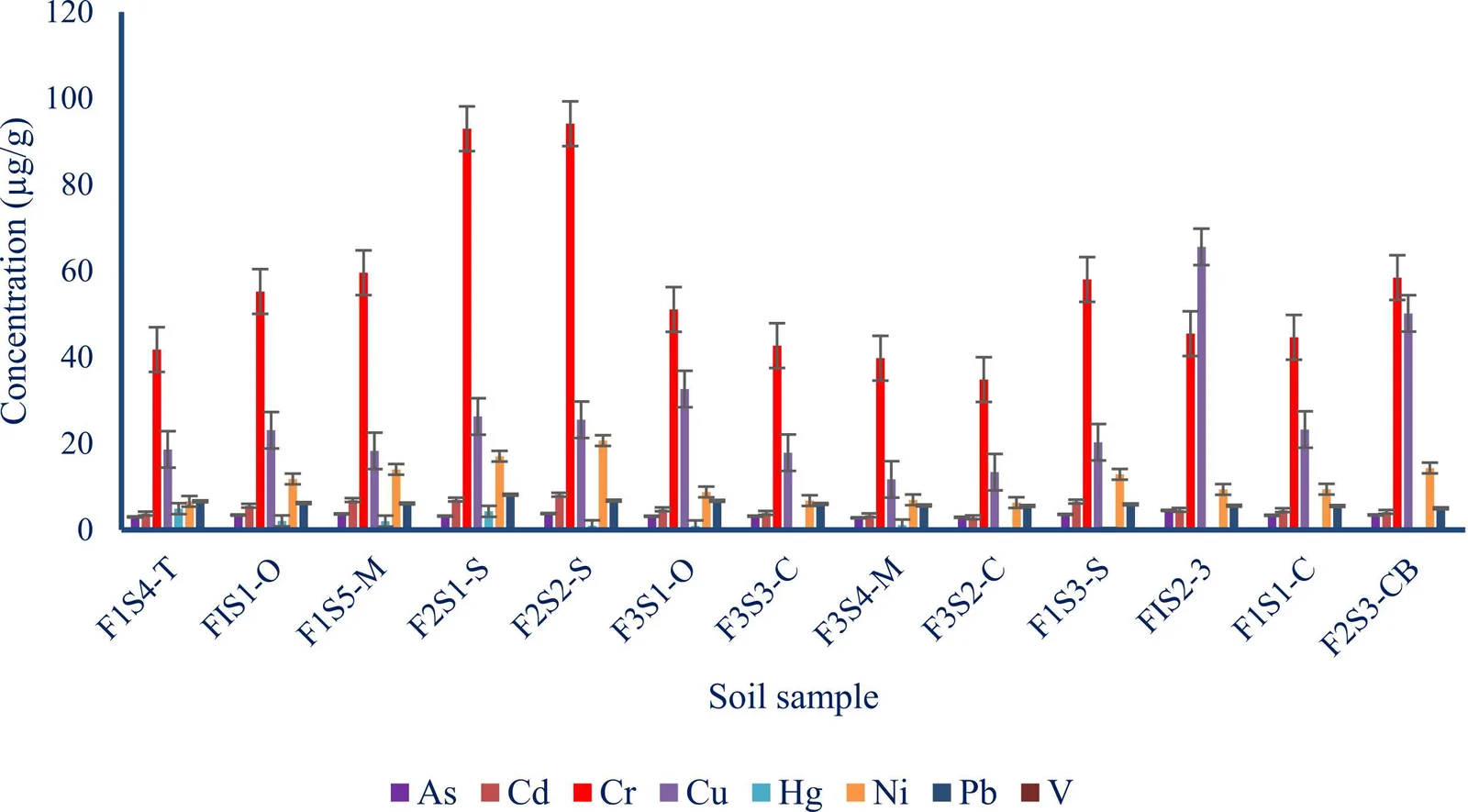
Total concentrations of trace elements in soil during low flow season.

#### 3.5.2 The total concentrations of trace elements in soil during high flow season

To characterize the soil associated with vegetable production during the high flow season, seven corresponding cultivation soil samples were analysed. The total concentrations of all the selected trace elements measured in these samples are presented in Fig. 8.

**Fig. 8:**
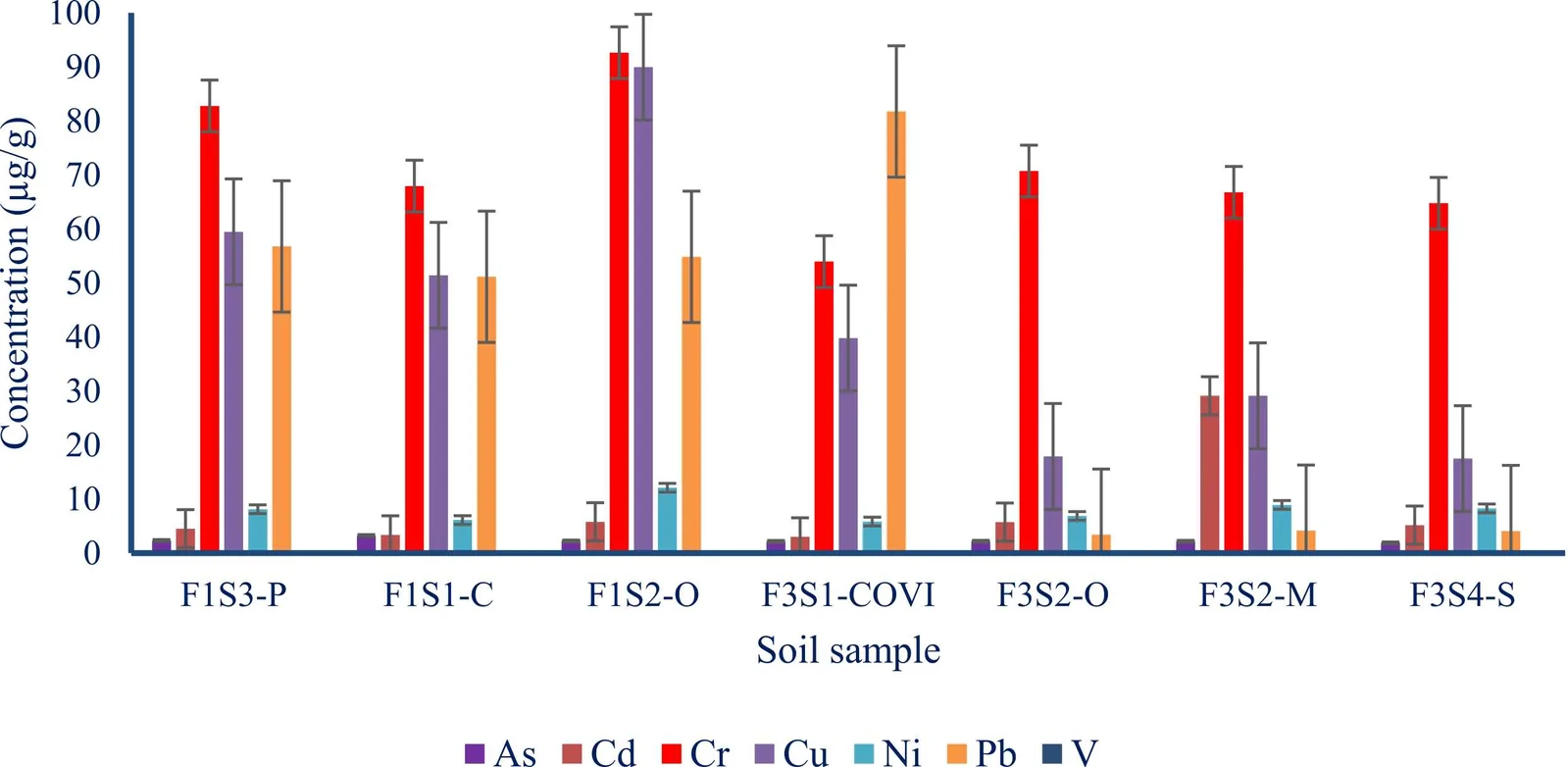
Total concentrations of trace elements in soil during high flow season.

During high flow season, Cd concentrations ranged from 3.01 to 29.12 µg/g, with a pronounced maximum at F3S2-M. Chromium concentrations ranged from 54.0 to 92.6 µg/g and were generally higher than those observed at most low-flow sampling locations. Copper varied considerably, reaching 89.9 µg/g at F1S2-O, while Pb reached 81.7 µg/g at F3S1-COVI. The pronounced variability observed for Cd, Cu and Pb indicates that trace element distribution within the agricultural soils was spatially heterogeneous this season.

Similar observations regarding the occurrence of trace elements across agricultural soil-crop systems have been reported by Zondo [20], who investigated soils and root vegetables from small-scale farms in KwaZulu-Natal. Trace elements were detected in both soils and different plant tissues, while bioaccumulation factors indicated transfer of selected elements from soils into plant tissues. This provides relevant South African context for considering agricultural soils as part of an integrated soil-crop exposure system rather than as an isolated environmental compartment. Statistical analysis showed that seasonal variations in Cd, Cr, Cu, Ni, and V were not significant (p > 0.05), while variations in As and Pb were statistically significant (p < 0.05) at a 95% confidence level. These trends may be attributed to increased rainfall and runoff during the high-flow season, as well as the application of organic fertilizers, which enhance soil fertility but may also contribute to the accumulation of trace elements [63], [64].

#### 3.5.3 Comparison of trace elements total concentrations in soil during low and high flow seasons

Trace elements concentration in soil used for cultivating the 13 vegetable sample were compared with the Canadian Soil Quality Guidelines (SQGs) (Table 9) [65].

**Table 9:** Canadian soil quality guidelines for selected trace elements.

Analyte	CCME	Maximum Concentrations	
	SQG (µg/g)	Low flow season (µg/g)	High flow season (µg/g)
As	12	4.52	3.25
Cd	1.4	8.15	29.1
Cr	64	94.1	92.6
Cu	63	65.6	89.9
Pb	70	8.15	81.7
Hg	6.6	4.92	< 0.0387
Ni	50	20.7	12.1
V	130	0.149	0.145

Comparison with the adopted SQGs identified Cd as the most consistent trace element of concern (Table 9**)**. All 13 low flow season and all seven high flow season soil samples exceeded the Cd guideline of 1.4 µg/g. The low flow season mean concentration (5.11 µg/g) was significantly greater than the guideline, *t*(12) = 8.33, *p* < 0.001. Although the high flow season mean (8.11 µg/g) was also numerically greater, it did not differ significantly from the guideline (*p* = 0.106). This result reflects the pronounced variability and non-normal distribution of the high flow season Cd data, particularly the concentration of 29.1 µg/g at F3S2-M. Accordingly, the high-flow *t*-test should be interpreted cautiously, while the exceedance observed in every individual sample demonstrates the widespread occurrence of elevated Cd concentrations.

Chromium showed a more seasonally differentiated pattern. During low flow season, two of thirteen samples exceeded the SQG of 64 µg/g, whereas six of seven samples exceeded this value during high flow season. Nevertheless, neither the low flow season mean (55.3 µg/g; *p* = 0.118) nor the high flow mean (71.4 µg/g; *p* = 0.175) differed significantly from the guideline. Copper and Pb exceedances were more localised, Cu exceeded its SQG at one sampling site during each season, while Pb exceeded its guideline at one site during high flow season. In contrast, As, Ni and V remained below their respective SQGs across the sampled agricultural soils. Collectively, these findings indicate widespread Cd enrichment, more extensive Cr exceedances during high flow season, and comparatively localised enrichment of Cu and Pb.

These findings are consistent with previous studies reporting trace element enrichment in soils from both international and South African settings. Luo et al. [66] reported elevated Cd concentrations exceeding the Dutch action value in soils surrounding an e-waste processing area in China, while Kapwata et al. [28] reported As concentrations exceeding the Canadian SQG in soils from Giyani, Limpopo Province, South Africa. Although the predominant trace elements differed among the study areas, these findings demonstrate the spatial variability of soil contamination and the influence of local environmental and anthropogenic conditions. In the present study, the widespread Cd exceedances, together with the more extensive Cr exceedances during high flow season and localised Cu enrichment, highlight the need for continued monitoring of agricultural soils to assess temporal changes in trace element accumulation and their potential implications in the food chain.

The predominance of Cd and Cr in agricultural soils is particularly relevant when considered alongside the vegetable results of the present study. However, elevated total soil concentrations do not necessarily indicate equivalent availability for plant uptake because trace element mobility and bioavailability are influenced by chemical speciation, soil physicochemical properties and crop-specific uptake mechanisms. Therefore, the concurrent occurrence of trace elements in soil and edible vegetables should not be interpreted as evidence of direct soil-to-plant transfer without determination of bioavailable fractions and crop-specific transfer factors. Nevertheless, the soil findings provide an important environmental context for the subsequent assessment of trace element accumulation in edible vegetables and the associated dietary health risks.

### 3.6 Health risk assessment

#### 3.6.1 Determination of the non-carcinogenic risk

In assessing the health risk assessment for both adults and children in Lephalale who consumes the contaminated vegetables, the THQ and HI were applied to evaluate the non-carcinogenic risks associated with their intake [53]. The THQ values for adult and children’s populations in Lephalale were calculated for both seasons and the results are presented in Fig. 9 to Fig. 12.

**Fig. 9:**
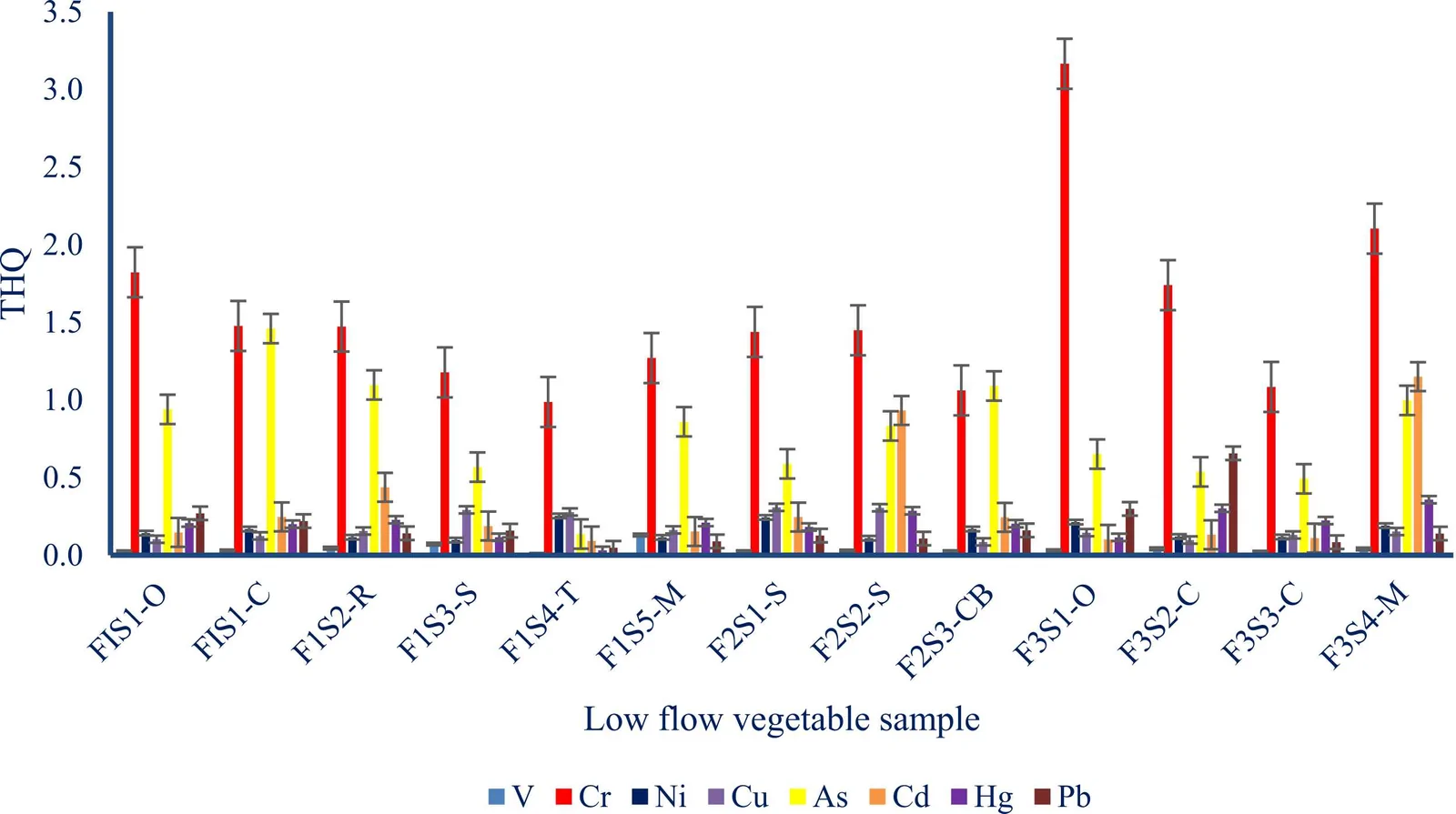
Adult THQ values during low flow season.

As illustrated in Fig. 9, Cr was the predominant contributor to non-carcinogenic risk among adults. THQ values exceeded 1 for almost all vegetables, reaching a maximum of 3.17 for onion (F3S1-O), only tomato (F1S4-T) remained marginally below the 1 (THQ = 0.987). Arsenic also exceeded the threshold in chomolia (F1S1-C), rape (F1S2-R) and cabbage (F2S3-CB), while Cd exceeded 1 only in Mo’china (F3S4-M; THQ = 1.15). In contrast, adult THQs for Cu, Pb, Hg, Ni and V remained below 1. These results indicate that Cr was the most consistent contributor to estimated adult non-carcinogenic risk during this season, whereas the contributions of As and Cd were more vegetable specific.

During high flow season, THQs shifted considerably. Chromium remained consistently above the threshold for adults across all vegetable samples and reached a maximum THQ of 4.33 in onion (F3S2-O). Nickel and Pb became particularly important in chomolia (F1S2-Chomolia) and covi (F3S2-Covi), with Ni THQs of 2.88 and 4.61 and Pb THQs of 1.17 and 4.21, respectively. Covi additionally exceeded the threshold for As (1.53) and Cu (1.11), whereas Cd, Hg and V remained below 1 in all vegetables for this season (Fig. 10**)**. Thus, while Cr remained a consistent contributor across seasons, this results demonstrate that the relative importance of other trace elements was strongly dependent on vegetable type and seasonal concentration patterns.

**Fig. 10:**
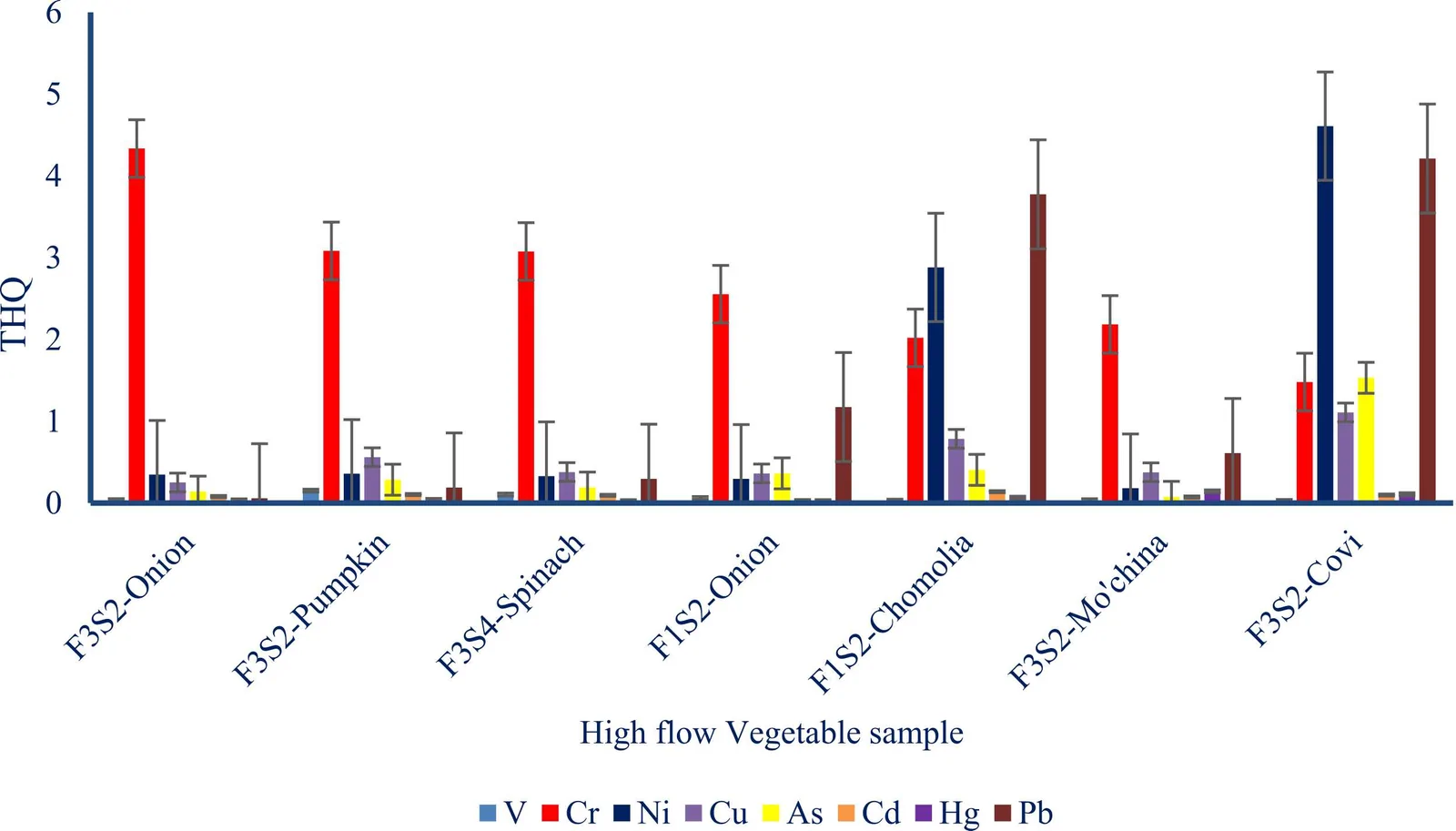
Adult THQ values during high flow season.

Overall, THQ values observed during the high-flow season were notably higher than those from the low-flow season, suggesting increased exposure risk due to seasonal variations. Among all vegetables, covi (F3S2-Covi) presented the highest cumulative risk to the adult population. These findings are consistent with those of Duan et al. [67], who identified Cr as a major contributor to elevated THQ values in both adults and children populations. In the current study, Cr similarly exhibited the highest THQ values in almost all vegetable samples, underscoring its critical role in health risk assessment.

The THQs were substantially greater for children. Chromium exceeded the threshold in all vegetables and reached a maximum THQ of 15.1 in onion (F3S1-O). Exceedances were also observed for As in most vegetables and, depending on the vegetable sample, Cd, Cu, Pb, Ni and Hg exceeded 1. However, V remained below 1 throughout (Fig. 11). The broader range of THQ exceedances among children demonstrates that the same vegetable concentrations can result in markedly different estimated risks between population groups because exposure is evaluated relative to body weight.

**Fig. 11:**
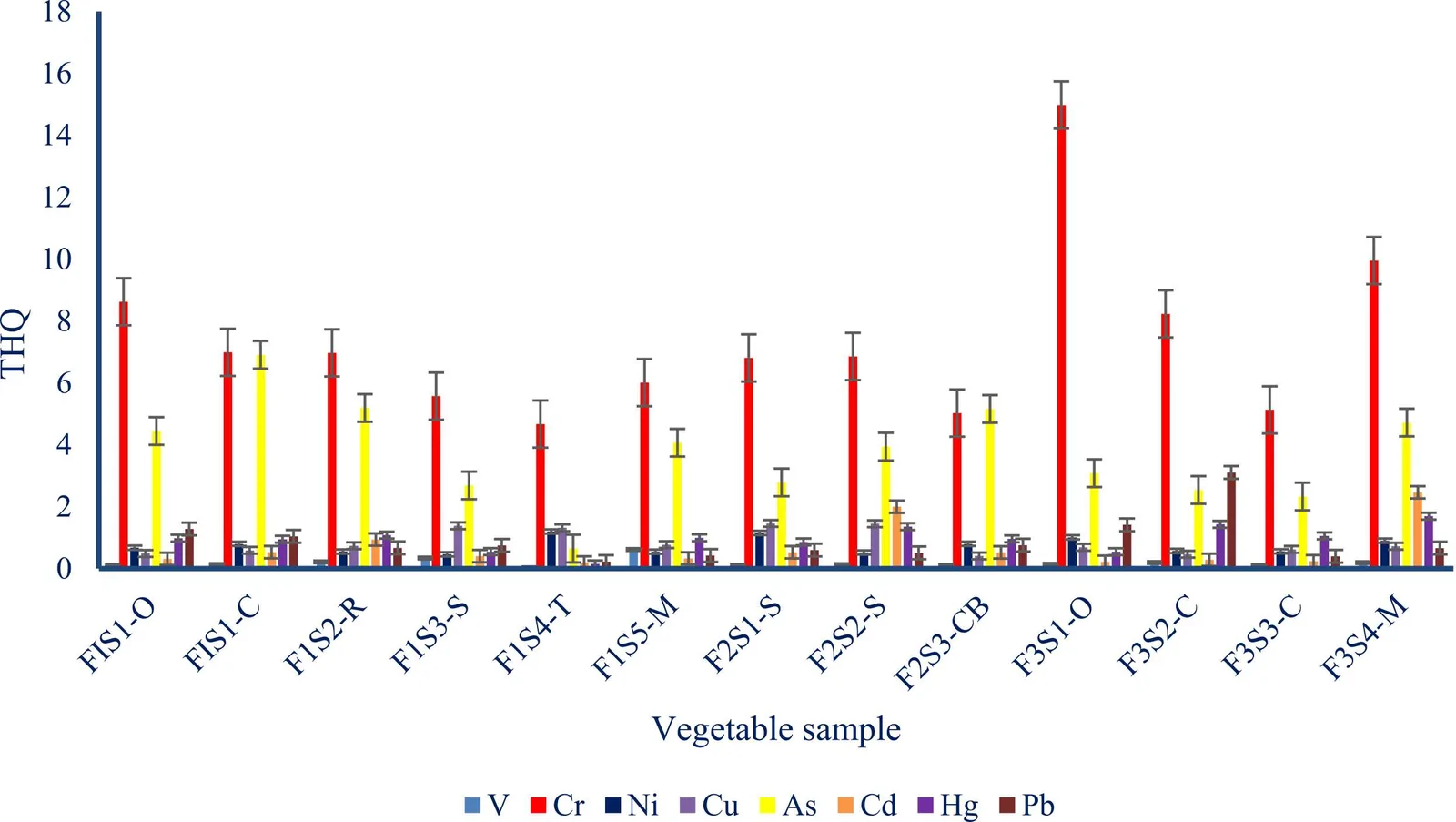
Children THQ values during low flow season.

This pattern was amplified in children. Chromium and Cu produced THQ values above 1 in vegetables across this season, while As and Pb exceeded the threshold in several samples. Most notably, Ni reached a THQ of 21.8 in covi (F3S2-Covi), indicating the highest THQ reported for children in this season (Fig. 12**)**. In contrast, Cd, Hg and V remained below the threshold in all vegetables. The pronounced Ni related risk associated with covi is consistent with its exceptionally high Ni concentration during this season, when this vegetable contained 54.7 µg/g Ni.

**Fig. 12:**
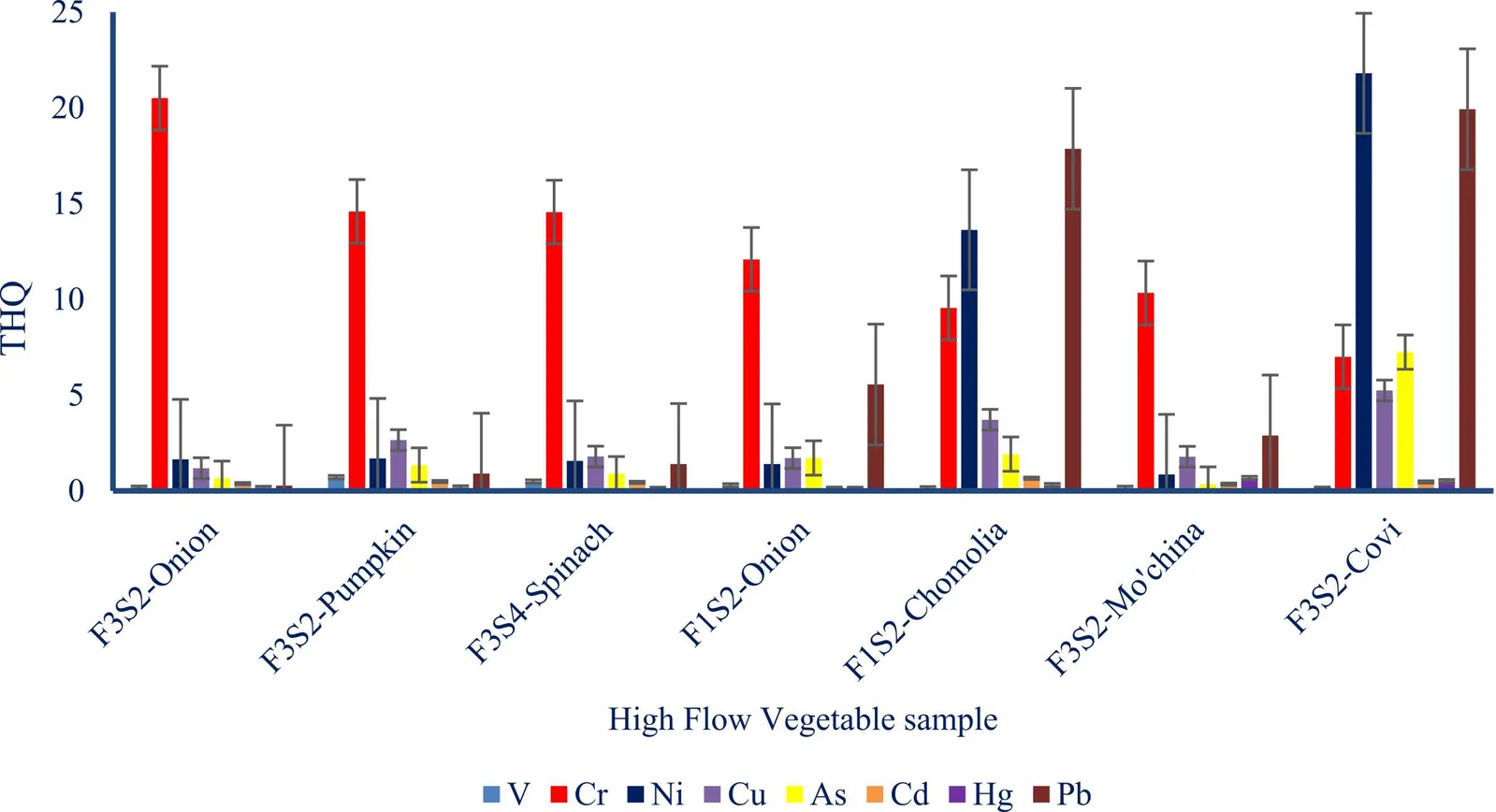
Children THQ values during high flow season.

The prominence of Cr across both seasons is supported by previous dietary-risk studies in which Cr contributed substantially to non-carcinogenic risk associated with consumption of trace element contaminated foods. The present results further demonstrate that risk cannot be inferred from trace element concentrations alone because the magnitude of the THQ depends on both exposure and element-specific toxicity parameters. Moreover, the substantially greater THQs estimated for children are consistent with those reported by Mantashloo et al. [68] reporting greater non-carcinogenic risk in children than adults attributed to higher intake rate per unit body weigh in children compared to adults. Similarly, Mamaregane et al. [69] reported substantially greater non-carcinogenic risk estimates for children than adults. The HI was determined. This metric represents the cumulative effect of all analysed trace elements within each vegetable sample. The calculated HI values for the low flow and high flow seasons are presented in Fig. 13 (low flow season) and Fig. 14 (high flow season). For each vegetable, the HI was computed by calculating the sum of concentrations of all the analytes presents in each vegetable sample.

**Fig. 13:**
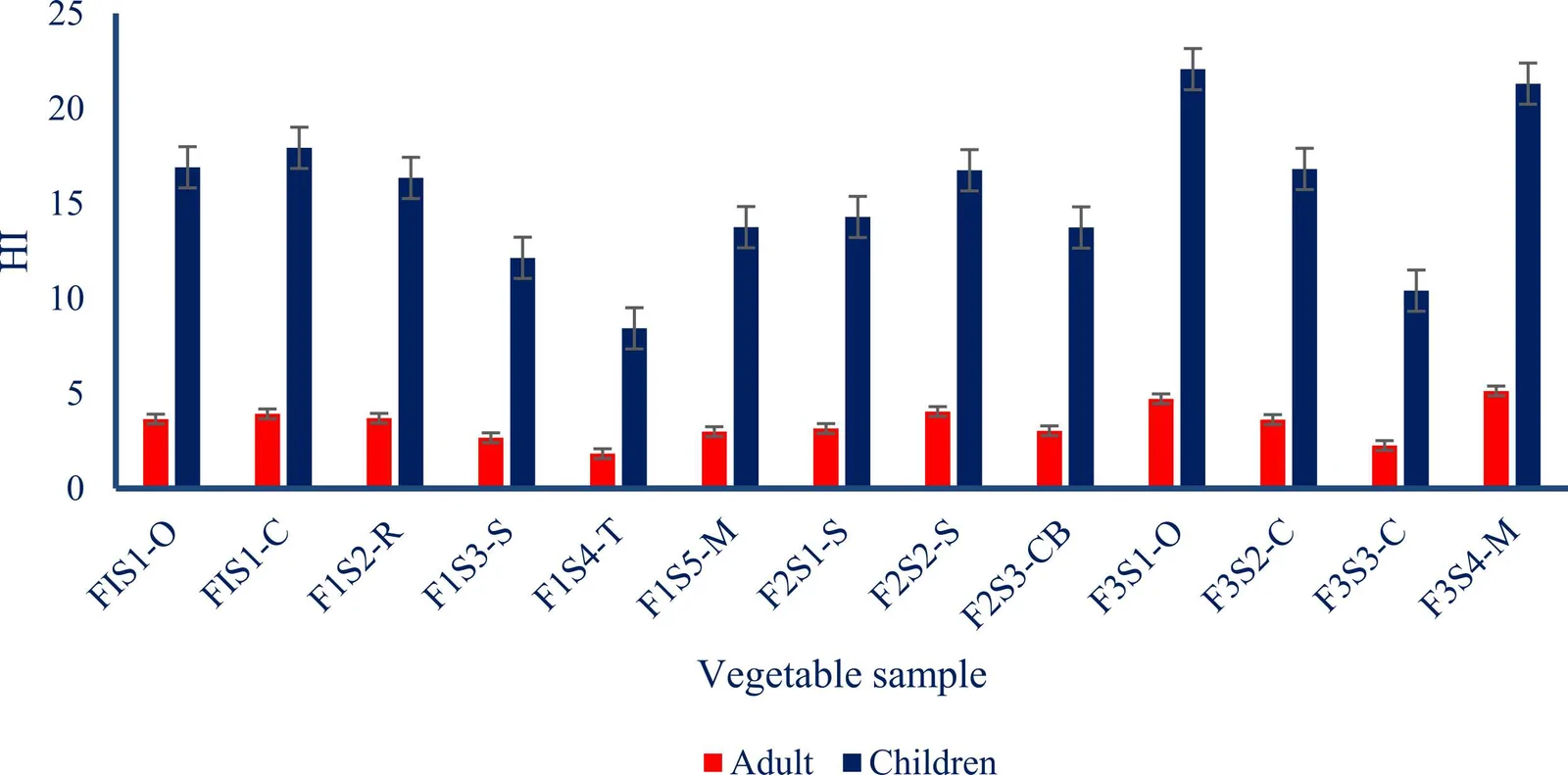
The HI values for adult and children population during low flow season.

**Fig. 14:**
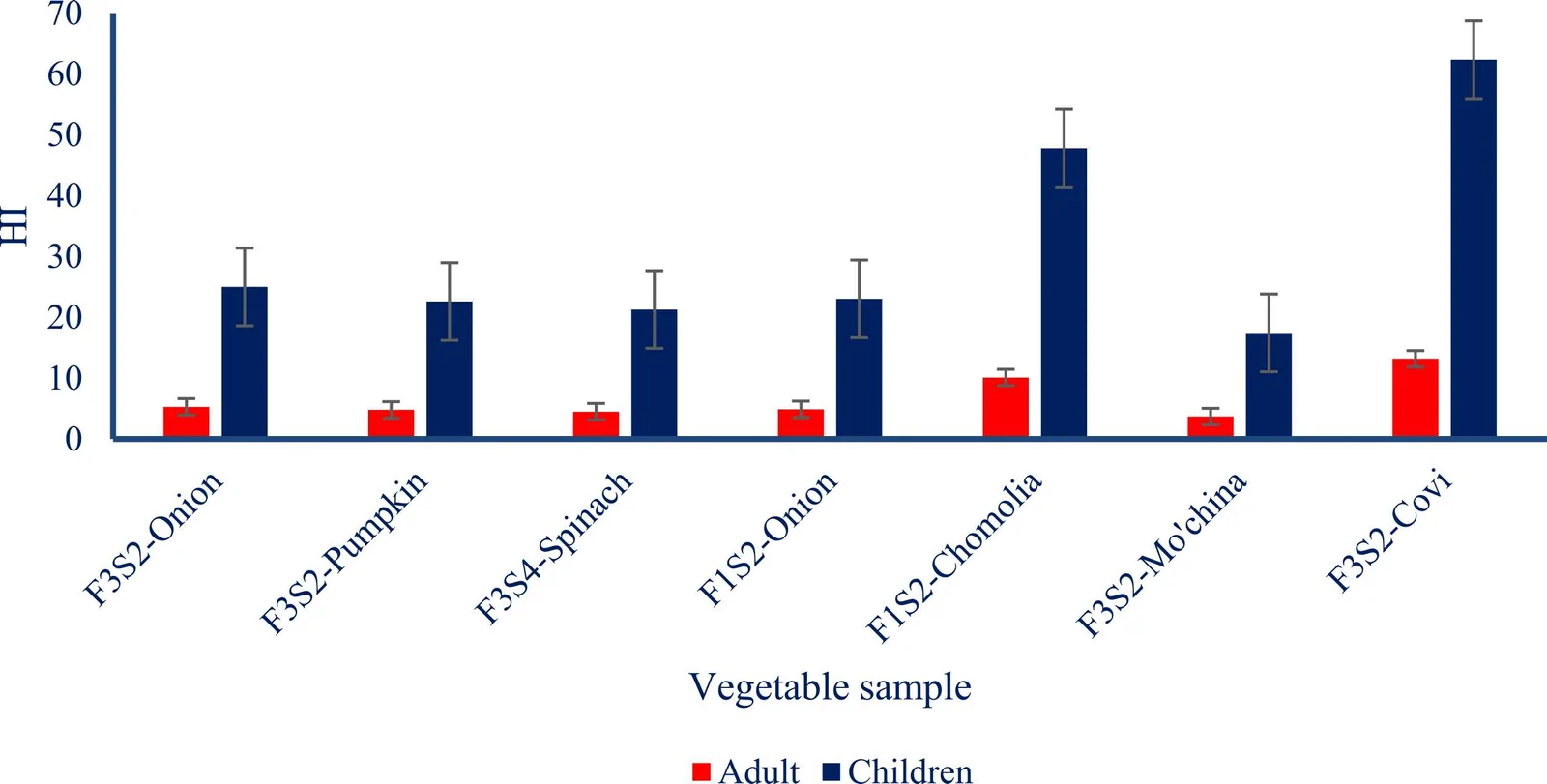
The HI values for adult and children population during high flow season.

The calculated HI values exceeded 1 for both adults and children in all evaluated vegetables during both seasons**,** indicating that combined exposure to the investigated trace elements may be of concern. During low flow season, the highest HI occurred in Mo’china (F3S4-M), reaching 5.13 for adults and 21.3 for children. During high flow season, covi (F3S2-Covi) produced substantially higher maximum values of 13.2 and 62.3 for adults and children, respectively.

The elevated HI values (Fig. 13 **and** Fig. 14**)** are particularly important because several vegetables for which individual trace elements had THQ values below 1, nevertheless produced a cumulative HI above the threshold. This demonstrates the importance of considering simultaneous dietary exposure to multiple trace elements rather than assessing each element independently. Similar findings have been reported by Pavlikova et al. [70] in vegetable risk assessments in which cumulative HI values above 1 indicated potential non-carcinogenic effects associated with combined exposure. The authors reported HI values above 1 for vegetables cultivated in trace element contaminated soils, with Cd, Pb and Cr making important contributions to cumulative risk.

Overall, the non-carcinogenic assessment identifies children as the more highly exposed population group and Cr as the most consistent individual contributor across seasons**,** while Ni, Pb, As and Cu became particularly important for selected vegetables during high flow season. The consistently elevated HI values further indicate that the potential health concern arises from combined dietary exposure to multiple trace elements, rather than from a single trace element alone. These findings support prioritising routine surveillance of trace elements in locally produced vegetables and targeted assessment of vegetables contributing most strongly to cumulative exposure, particularly where they are consumed frequently by children.

#### 3.6.2 Determination of the carcinogenic risk

The carcinogenic health risk associated with vegetable consumption was assessed using the ILCR for As, Cr, Cd, and Pb in adults and children. The ILCR values obtained during the low- and high-flow seasons are presented in Table 10, Table 11, respectively.

**Table 10:** Cancer risk associated with consumption of Vegetable sample during low flow season.

	As		Cr		Cd		Pb	
Sample	Adult	Children	Adult	Children	Adult	Children	Adult	Children
F1S1-O	${2.78\;\times 10}^{-5}$	${1.37\times 10}^{-4}$	${6.16\times 10}^{-1}$	${3.14\times 10}^{0}$	${2.09\;\times 10}^{-4}$	${2.03\;\times 10}^{-3}$	${2.01\;\times 10}^{-2}$	${1.19\times 10}^{-1}$
F1S1-C	${5.67\times 10}^{-5}$	${2.08\times 10}^{-4}$	${4.38\times 10}^{-1}$	${2.16\times 10}^{0}$	${2.72\times 10}^{-4}$	${1.34\times 10}^{-3}$	${7.81\times 10}^{-2}$	${3.85\times 10}^{-1}$
F1S2-R	${3.77\times 10}^{-5}$	${1.86\times 10}^{-4}$	${4.37\times 10}^{-1}$	${2.16\times 10}^{0}$	${2.46\times 10}^{-4}$	${1.21\times 10}^{-3}$	${1.22\times 10}^{-1}$	${6,01\times 10}^{-1}$
F1S3-S	${7.22\times 10}^{-5}$	${3.56\times 10}^{-4}$	${3.63\times 10}^{-1}$	${1.79\times 10}^{0}$	${7.84\times 10}^{-5}$	${3.87\times 10}^{-4}$	${4.81\times 10}^{-1}$	${2.37\times 10}^{0}$
F1S4-T	${8.07\times 10}^{-5}$	${3.98\times 10}^{-4}$	${2.87\times 10}^{-1}$	${1.42\times 10}^{0}$	${3.64\times 10}^{-4}$	${1.80\times 10}^{-3}$	${1.55\times 10}^{0}$	${7.63\times 10}^{0}$
F1S5-M	${1.50\times 10}^{-5}$	${7.42\times 10}^{-5}$	${3,10\times 10}^{-1}$	${1.53\times 10}^{0}$	${1.94\times 10}^{-4}$	${9.57\times 10}^{-4}$	${2.51\times 10}^{-1}$	${1.24\times 10}^{0}$
F2S1-S	${3.05\times 10}^{-4}$	${1.50\times 10}^{-3}$	${2.10\times 10}^{-1}$	${1.04\times 10}^{0}$	${2.57\times 10}^{-4}$	${1.27\times 10}^{-3}$	${1.73\times 10}^{0}$	${8.51\times 10}^{0}$
F2S2-S	${3.05\times 10}^{-4}$	${3.05\times 10}^{-4}$	${3.05\times 10}^{-4}$	${3.05\times 10}^{-4}$	${3.05\times 10}^{-4}$	${3.05\times 10}^{-4}$	${3.05\times 10}^{-4}$	${3.05\times 10}^{-4}$
F2S3-CB	${3.05\times 10}^{-4}$	${3.05\times 10}^{-4}$	${3.05\times 10}^{-4}$	${3.05\times 10}^{-4}$	${3.05\times 10}^{-4}$	${3.05\times 10}^{-4}$	${3.05\times 10}^{-4}$	${3.05\times 10}^{-4}$
F3S1-O	${3.05\times 10}^{-4}$	${3.05\times 10}^{-4}$	${3.05\times 10}^{-4}$	${3.05\times 10}^{-4}$	${3.05\times 10}^{-4}$	${3.05\times 10}^{-4}$	${3.05\times 10}^{-4}$	${3.05\times 10}^{-4}$
F3S2-C	${3.05\times 10}^{-4}$	${3.05\times 10}^{-4}$	${3.05\times 10}^{-4}$	${3.05\times 10}^{-4}$	${3.05\times 10}^{-4}$	${3.05\times 10}^{-4}$	${3.05\times 10}^{-4}$	${3.05\times 10}^{-4}$
F3S3-C	${3.05\times 10}^{-4}$	${3.05\times 10}^{-4}$	${3.05\times 10}^{-4}$	${3.05\times 10}^{-4}$	${3.05\times 10}^{-4}$	${3.05\times 10}^{-4}$	${3.05\times 10}^{-4}$	${3.05\times 10}^{-4}$
F3S4-M	${3.05\times 10}^{-4}$	${3.05\times 10}^{-4}$	${3.05\times 10}^{-4}$	${3.05\times 10}^{-4}$	${3.05\times 10}^{-4}$	${3.05\times 10}^{-4}$	${3.05\times 10}^{-4}$	${3.05\times 10}^{-4}$

**Table 11:** Cancer risk associated with consumption of Vegetable sample during high flow season.

	As		Cr		Cd		Pb	
Sample	Adult	Children	Adult	Children	Adult	Children	Adult	Children
F3S2-Onion	${2.78\;\times 10}^{-5}$	${1.37\times 10}^{-4}$	${6.16\times 10}^{-1}$	${3.14\times 10}^{0}$	${2.09\;\times 10}^{-4}$	${2.03\;\times 10}^{-3}$	${2.01\;\times 10}^{-2}$	${1.19\times 10}^{-1}$
F3S2-Pumpkin	${5.67\times 10}^{-5}$	${2.08\times 10}^{-4}$	${4.38\times 10}^{-1}$	${2.16\times 10}^{0}$	${2.72\times 10}^{-4}$	${1.34\times 10}^{-3}$	${7.81\times 10}^{-2}$	${3.85\times 10}^{-1}$
F3S4-Spinach	${3.77\times 10}^{-5}$	${1.86\times 10}^{-4}$	${4.37\times 10}^{-1}$	${2.16\times 10}^{0}$	${2.46\times 10}^{-4}$	${1.21\times 10}^{-3}$	${1.22\times 10}^{-1}$	${6,01\times 10}^{-1}$
F1S2-Onion	${7.22\times 10}^{-5}$	${3.56\times 10}^{-4}$	${3.63\times 10}^{-1}$	${1.79\times 10}^{0}$	${7.84\times 10}^{-5}$	${3.87\times 10}^{-4}$	${4.81\times 10}^{-1}$	${2.37\times 10}^{0}$
F1S2-Chomoli	${8.07\times 10}^{-5}$	${3.98\times 10}^{-4}$	${2.87\times 10}^{-1}$	${1.42\times 10}^{0}$	${3.64\times 10}^{-4}$	${1.80\times 10}^{-3}$	${1.55\times 10}^{0}$	${7.63\times 10}^{0}$
F3S2-Mo'china	${1.50\times 10}^{-5}$	${7.42\times 10}^{-5}$	${3,10\times 10}^{-1}$	${1.53\times 10}^{0}$	${1.94\times 10}^{-4}$	${9.57\times 10}^{-4}$	${2.51\times 10}^{-1}$	${1.24\times 10}^{0}$
F3S2-Covi	${3.05\times 10}^{-4}$	${1.50\times 10}^{-3}$	${2.10\times 10}^{-1}$	${1.04\times 10}^{0}$	${2.57\times 10}^{-4}$	${1.27\times 10}^{-3}$	${1.73\times 10}^{0}$	${8.51\times 10}^{0}$

During the low-flow season, Cr related ILCR values remained consistently elevated across the vegetable samples (Table 10). The highest values occurred in F3S1-O, reaching ${4.50\;\times 10}^{-1}$ in adults and 2.22 in children, while children's Cr related ILCR exceeded 1 in eight of the thirteen samples. Lead related risk was lower than the extreme values recorded during low flow season but remained elevated in F3S2-C, where ILCR reached ${2.69\;\times 10}^{-1}$ in adults and 1.33 in children. In contrast, the highest Cd related ILCR occurred during high flow season in F3S4-M, reaching ${1.36\times 10}^{-3}$ in adults and ${6.73\;\times 10}^{-3}$ in children. The maximum As related values were recorded in F1S1-C at ${2.90\;\times 10}^{-4}$ and ${1.43\;\times 10}^{-3}$ for adults and children, respectively.

During the high-flow season, Cr and Pb accounted for the greatest estimated carcinogenic risks, although substantial variation was observed among vegetable samples (Table 11). The highest Cr-related ILCR occurred in F3S2-Onion, reaching ${6.16\;\times 10}^{-1}$ in adults and 3.04 in children. Lead produced the highest sample-specific risks, particularly in F3S2-Covi (1.73 in adults and 8.51 in children) and F1S2-Chomolia (1.55 and 7.63, respectively.

Comparison between seasons revealed distinct element-specific patterns. Chromium was the most consistently elevated contributor across both seasons, although its maximum ILCR increased from low to high flow season. This finding is in harmony with Alegbe et al. [71], who identified Cr as the principal contributor to carcinogenic risk among Cr, Cd, and Pb in vegetables. Rahati et al. [33], similarly reported carcinogenic risk associated with dietary Cr exposure, although at substantially lower levels. South African evidence has also identified Cr as an important contributor to lifetime cancer risk associated with consumption of trace element contaminated fruits and vegetables [72]. The comparatively high Cr-related ILCR values in the present study may be associated with the elevated Cr concentrations measured in the vegetables, although differences in vegetable type, dietary exposure assumptions, and toxicity parameters should be considered when comparing risk estimates among studies [51], [70], [72].

Interpretation of the Cr related ILCR requires caution because the present study quantified total Cr without differentiating between Cr(III) and Cr(VI). Previous investigation of vegetables from the Mokolo River area reported Cr(III) concentrations of 374–17,400 ng/g compared with 197–1180 ng/g for Cr(VI) [10]. Furthermore, species specific assessment identified Cr(VI) as the Cr species associated with the greatest estimated carcinogenic risk [10]. Accordingly, the Cr related ILCR values reported here should be interpreted as modelled estimates based on the cancer slope factor applied to total Cr rather than as evidence that all Cr measured in the vegetables occurred as Cr(VI).

Lead exhibited the strongest seasonal contrast, with the maximum ILCR increasing from ${2.69\;\times 10}^{-1}$ to ${1.73\;\times 10}^{0}$ to in adults and from 1.33 to 8.51 in children between the low and high-flow seasons, respectively. The exceptionally high low-flow values were concentrated in specific vegetable samples rather than occurring uniformly across the dataset. This contrasts with Alegbe et al. [71], who reported substantially lower Pb related cancer risks and identified Pb as the lowest contributor among Cr, Cd, and Pb. These differences may partly be attributed to variation in trace element accumulation among vegetable species, as previous studies have demonstrated considerable differences in Cd, Cr, and Pb accumulation and associated health risks among crops [70]. The present results therefore indicate pronounced vegetable and sampling site specific variation in Pb related dietary risk.

Cadmium exhibited a different seasonal pattern, with the maximum children's ILCR decreasing from ${6.73\;\times 10}^{-3}$ during low flow season to ${1.80\;\times 10}^{-3}$ during high flow season. Although its contribution was lower than the highest risks associated with Cr and Pb, Alegbe et al. [71] similarly identified Cd as an important contributor to carcinogenic risk from vegetable consumption. In contrast, As showed comparatively little difference in maximum ILCR between seasons, with children's values reaching ${1.43\;\times 10}^{-3}$ during low flow season and ${1.50\;\times 10}^{-3}$during high flow season. Previous investigation of vegetables from the Mokolo River area also demonstrated seasonal variation in As contamination [10]. These contrasting patterns indicate that seasonal variation in estimated carcinogenic risk was element specific rather than uniformly greater during either flow season.

The highest element specific ILCR values occurred in different vegetable samples, indicating considerable variation according to vegetable type and sampling location. Similar crop dependent differences in trace element accumulation and associated health risk have been reported among vegetable species [70]. Alegbe et al. [71], likewise observed differences in carcinogenic risk among lettuce, spring onion, and cabbage. These findings suggest that variation in trace element accumulation among vegetable species may contribute to the observed risk profiles. However, the physiological mechanisms governing uptake were not specifically investigated in the present study.

A consistent finding across both seasons and all four trace elements was the higher ILCR estimated for children than adults. Previous research involving vegetables from the Mokolo River area similarly reported that the probability of higher cancer risk associated with vegetable consumption was approximately 4.73 times greater in children than adults [10]. Greater carcinogenic risks among children have also been reported for trace-element exposure through fruits and vegetables in Johannesburg [72]. This difference is consistent with the age specific exposure parameters incorporated into the risk assessment, particularly dietary intake relative to body weight [10]. The ILCR values therefore represent modelled lifetime carcinogenic risks under the assumed exposure conditions and should not be interpreted as predictions of actual cancer occurrence.

### 3.7 Advantages, limitations and future research directions

A major strength of this study is its integrated assessment of trace elements across water, sediment, agricultural soil and locally cultivated vegetables within the Mokolo River catchment under contrasting low- and high-flow seasons. The inclusion of multiple environmental matrices, together with non-carcinogenic and carcinogenic health risk assessments for adults and children, provides a broader evaluation of environmental occurrence and potential human exposure than assessment of individual matrices alone. This approach is particularly relevant because the findings demonstrate that compliance with water-quality guidelines does not necessarily reflect trace elements occurrence within other components of the surrounding agroecosystem.

Nevertheless, several limitations should be considered when interpreting the findings. Sampling was restricted to two seasonal periods and therefore may not capture longer-term temporal variability in trace element concentrations. Furthermore, total trace element concentrations were determined rather than their chemical species or bioavailable fractions, limiting conclusions regarding mobility and bioavailability. Although trace elements were quantified in both agricultural soils and vegetables, direct soil-to-plant transfer cannot be established without determination of bioavailable soil fractions and crop-specific transfer factors. The deterministic human health risk assessment is also dependent on the exposure parameters and toxicity reference values applied and therefore represents a screening-level estimate of potential risk rather than a direct prediction of adverse health outcomes.

Future studies should incorporate repeated sampling over multiple years to establish longer-term temporal trends and should investigate speciation of trace elements, bioavailability and soil-to-plant transfer factors to better characterise environmental exposure pathways. Expanded dietary exposure assessments incorporating vegetable consumption patterns representative of the local population would further strengthen estimates of human exposure. Continued monitoring of river water, sediment, agricultural soils and edible crops is recommended, particularly in areas prone to pollution and for vegetables associated with elevated concentrations of trace elements and high health-risk estimates.

## 4 Conclusion

This study provides an integrated assessment of trace element contamination in water, sediment, soil and vegetable compartments of Mokolo River catchment and highlights its implications for food safety and human health. Importantly, the findings demonstrate that compliance of river water with established water quality guidelines does not necessarily indicate the absence of contamination within the broader agroecosystem. Elevated concentrations were observed in other environmental compartments, including Cd and Cr in agricultural soils and selected trace elements in sediments and edible vegetables. Seasonal and spatial variations were also evident, demonstrating the importance of considering changes in environmental conditions when evaluating trace element distribution and potential dietary exposure.

The study contributes to the understanding of trace element contamination in the Lephalale area by linking environmental occurrence with dietary exposure through locally cultivated vegetables. The non-carcinogenic and carcinogenic risk assessments identified potential health concerns associated with vegetable consumption, with children consistently exhibiting greater potential health risks than adults. Chromium was the prominent contributor to the estimated carcinogenic risks in both seasons, while Pb showed pronounced vegetable and site-specific contributions, particularly during high flow season. These findings demonstrate that potential health risks were dependent on trace element, vegetable type, sampling site, season, and population group rather than being uniformly distributed across the study area.

Therefore, the findings support an integrated monitoring approach that extends beyond water quality to include sediments, agricultural soils, and edible crops in mining affected and agriculturally active areas. Such monitoring could support evidence based agricultural management, food safety interventions, and regulatory decision making. Future studies should investigate long term temporal trends, trace element speciation and bioavailability, and soil to plant transfer mechanism to better characterise environmental exposure pathway and associated human risks.

## CRediT authorship contribution statement

**Pertunia Ramolwetsi Dikotla:** Writing – original draft, Visualization, Validation, Methodology, Investigation, Formal analysis, Data curation, Conceptualization. **Abayneh Ataro Ambushe:** Writing – review & editing, Visualization, Validation, Supervision, Resources, Project administration, Methodology, Investigation, Funding acquisition, Conceptualization.

## Funding

This work was supported and funded by the 10.13039/501100004424**Water Research Commission** **(WRC) of South Africa**, **Project Number** **C2022/2023–00933** and the 10.13039/501100001321**National Research Foundation** **(NRF) of South Africa**, **Grant Number** **CSUR240406212531** as well as the **NRF** general scholarship **Grant UID of** **141598**.

## Declaration of Competing Interest

The authors declare the following financial interests/personal relationships which may be considered as potential competing interests: Abayneh Ataro Ambushe reports financial support was provided by Water Research Commission. Abayneh Ataro Ambushe reports financial support was provided by National Research Foundation. If there are other authors, they declare that they have no known competing financial interests or personal relationships that could have appeared to influence the work reported in this paper.

## Data Availability

Data will be made available on request.
